# Identification and interrogation of the gene regulatory network of *CEBPA*-double mutant acute myeloid leukemia

**DOI:** 10.1038/s41375-022-01744-5

**Published:** 2022-11-04

**Authors:** Assunta Adamo, Paulynn Chin, Peter Keane, Salam A. Assi, Sandeep Potluri, Sophie G. Kellaway, Daniel Coleman, Luke Ames, Anetta Ptasinska, H. Ruud Delwel, Peter N. Cockerill, Constanze Bonifer

**Affiliations:** 1 Institute of Cancer and Genomic Sciences, University of Birmingham, B152TT Birmingham, UK; 2 Department of Hematology, Erasmus MC Cancer Institute, Rotterdam, The Netherlands

**Keywords:** Cancer genomics, Haematological cancer

## Abstract

Acute myeloid leukemia (AML) is a heterogeneous hematological malignancy caused by mutations in genes encoding transcriptional and epigenetic regulators together with signaling genes. It is characterized by a disturbance of differentiation and abnormal proliferation of hematopoietic progenitors. We have previously shown that each AML subtype establishes its own core gene regulatory network (GRN), consisting of transcription factors binding to their target genes and imposing a specific gene expression pattern that is required for AML maintenance. In this study, we integrate gene expression, open chromatin and ChIP data with promoter-capture Hi-C data to define a refined core GRN common to all patients with *CEBPA*-double mutant (*CEBPA*^*N/C*^) AML. These mutations disrupt the structure of a major regulator of myelopoiesis. We identify the binding sites of mutated C/EBPα proteins in primary cells, we show that C/EBPα, AP-1 factors and RUNX1 colocalize and are required for AML maintenance, and we employ single cell experiments to link important network nodes to the specific differentiation trajectory from leukemic stem to blast cells. Taken together, our study provides an important resource which predicts the specific therapeutic vulnerabilities of this AML subtype in human cells.

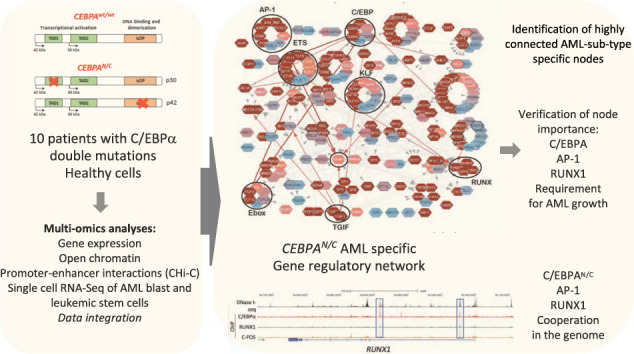

## Introduction

AML-causing genetic abnormalities that disrupt transcription factors (TFs) such as RUNX1 and C/EBPα [1, 2] interfere with hematopoietic differentiation. Different mutations lead to different disease phenotypes, gene expression profiles and clinical outcomes [3]. We previously defined the gene regulatory networks (GRNs) of seven AML subtypes including *t(8;21), Inv(16), FLT3-TKD, FLT3-ITD, NPM1, RUNX1*, biallelic *CEBPA*^*N/C*^ and *CEBPA* single mutations by collecting transcriptome, digital footprinting and chromatin conformation capture data [4]. We showed that each AML subtype is maintained by a distinct GRN which differs from that of normal myeloid progenitor cells, demonstrating that aberrant differentiation trajectories generated new cellular identities.

**Fig. 1:**
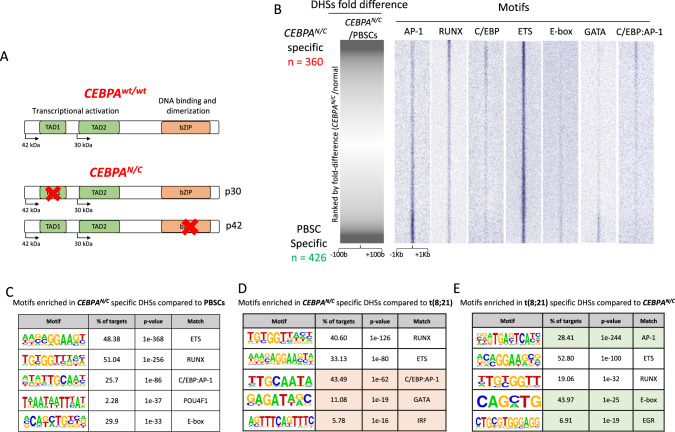
*CEBPA*^*N/C*^ patients form a distinct group that is related but not identical to t(8;21) AML. **A** Representation of the wild-type and mutated C/EBPα transcription factor. Transactivation (TAD) and dimerization/DNA-binding domains are highlighted. **B** Hypersensitive sites fold difference between *CEBPA*^*N/C*^ and PBSCs across a 200 bp window. Data are ranked by normalized tag counts of merged *CEBPA*^*N/C*^ peaks over merged PBSCs peaks. TF binding motifs projected against hypersensitive sites are plotted alongside. **C**–**E** Motifs enriched in *CEBPA*^*N/C*^ specific hypersensitive sites compared to PBSCs (**C**) and motif analysis in *CEBPA*^*N/C*^ (**D**) and t(8;21) (**E**) specific accessible chromatin regions. Motifs differentially enriched between the two AML subtypes are depicted in pink and green.

C/EBPα is essential for the generation of granulocyte-monocyte progenitors (GMPs) [5, 6]. The *CEBPA* gene is mutated in ~15% of all AMLs [7] with most mutations affecting either the N-terminal transactivation domain (TAD1, *CEBPA*^N^) or the C-terminal basic region leucine (bZIP) domain (*CEBPA*^C^) (Fig.1A) [8]. N-terminal mutations give rise to a premature stop codon, whereby internal re-initiation of translation results in the exclusive expression of the shorter C/EBPα-p30 isoform. C-terminal mutations alter the bZIP domain and disrupt C/EBPα DNA-binding and dimerization ability [9, 10]. About 50% of *CEBPA* mutant AMLs exhibit biallelic mutations with combined N-terminal and C-terminal lesions (*CEBPA*^*N/C*^) [11, 12].

Mouse models of *CEBPA* mutated AML showed that homozygous C-terminal mutations promote the expansion of pre-leukemic HSCs but impair myeloid differentiation, whereas N-terminal mutations do not impact on proliferation and preserve myeloid commitment. The combination of both mutations accelerates leukemogenesis and explains the *CEBPA*^*N/C*^ prevalence [13] whereby the sole expression of the p30 isoform is sufficient to develop AML [14]. A recent study identified genomic regions bound by p30 in a mouse model of *CEBPA*^N^ AML [15] but the binding pattern of p30 in C/EBPα^N/C^ patients is unknown.

GRNs represent the regulatory connections between TFs and their target genes [16]. GRNs inference has traditionally relied on gene expression perturbation studies [17] but, more recently, connections between genes and TFs were determined by measuring TF binding directly [4, 18, 19]. Previously, we constructed a basic GRN of *CEBPA*^*N/C*^ AML patients by linking distal cis-regulatory elements occupied by specific TF families to their rightful promoter using promoter-capture Hi-C data from other AML subtypes [4]. This work showed that the cellular identity of *CEBPA*^*C/N*^ is based on distinct regulatory relationships. However, the C/EBPα binding pattern in patient cells remained unknown, the preliminary GRN was based on only three patients, considered only distal regulatory elements, and was not based on *CEBPA*^*N/C*^-specific Hi-C data. In this study, we defined the complete core GRN of *CEBPA*^*N/C*^ AML by integrating multi-omics data from 10 patients. We identified *CEBPA*^*N/C*^ AML-specific cis-regulatory elements and TF modules, highlight AP-1, RUNX1 and C/EBPα as crucial factors driving AML maintenance and show that mutant C/EBPα and AP-1 factors colocalize with RUNX1 in chromatin. Our study therefore provides important insights into the biology of the disease in humans.

## Methods

Detailed methods can be found in the Supplementary information.

### Cell lines and cell line culture

Kasumi-1 and HEK 293T cells were purchased from the Deutsche Sammlung von Mikroorganismen und Zellkulturen. KO52 were obtained from the Japanese Collection of Research Bioresources Cell Bank. Kasumi-1 cells were cultured in RPMI-1640 (Sigma-Aldrich) supplemented with 10% fetal calf serum (FCS) (Gibco), 5% Penicillin-Streptomycin (Pen-Strep) (Sigma-Aldrich), 5% L-Glutamine (L-Glu) (Sigma-Aldrich). HEK 293T cells were cultured in DMEM supplemented with 10% FCS (Gibco), 5% Pen-Strep, 5% L-Glu. KO52 were cultured in Alpha MEM (Lonza) supplemented with 10% FCS (Gibco), 5% Pen-Strep, 5% L-Glu.

### Primary cell culture and isolation of hematopoietic stem/progenitor cells

*C/EBPA*^*N/C*^*-8* cells were cultured at the concentration of 1 × 10^6^ cell/mL in StemSpanTM Serum-Free Expansion Medium II (SFEMII) plus 10% StemSpanTM CD34+ Expansion Supplement and 175 nM UM171 (STEMCELL Technologies). All other patient cells were grown in SFEMII supplemented with 100 ng/mL thrombopoietin, 10 ng/mL FMS-like tyrosine kinase 3 ligand, 750 nM Stem Regenin 1 (SRI), 10 ng/mL Interleukin 3, 10 ng/mL human granulocyte/macrophage colony stimulating factor, 150 ng/mL Stem Cell Factor (all Peprotech) and UM729 (STEMCELL Technologies). Patient CD34+ or CD117+ progenitors were isolated with CD34 MicroBead Kit, human or CD117 MicroBead Kit, human (Miltenyi-Biotech) as described in [4].

### dnC/EBP and dnFOS lentivirus plasmids

The doxycycline inducible pCW57.1-dnC/EBP plasmid was generated using Gateway Gene Cloning (Thermo Fisher Scientific). Plasmids containing the dnC/EBP and dnFOS inserts were a gift from Charles Vinson, National Cancer Institute, Bethesda, USA [20]. The pCW57.1 containing the dnFOS insert was generated by Dr Sandeep Potluri (University of Birmingham) following the same protocol [21].

### RNA extraction, real-time PCR and RNA-seq library preparation

Total RNA was extracted with NucleoSpin RNA (Machery-Nagel) and was retrotranscribed using SuperScriptTM II Reverse Transcriptase kit (Thermo Fisher) and Oligo(dT) primers (Promega). Real-time PCR and RNA-Seq was performed as described in [4].

### DNase-seq and ATAC-seq

DHS mapping was performed as described in [4]. ATAC libraries were generated following the Omni-ATAC protocol from [22].

### Chromatin immunoprecipitation followed by sequencing (ChIP-seq)

Double protein–protein crosslinking followed by protein-DNA crosslinking with 0.25 M Di(N-succinimidyl) glutarate (DSG) (Sigma-Aldrich) for 45 min on rotation at RT was performed when immunoprecipitating chromatin with the c-FOS antibody, a single-crosslinking approach (protein-DNA crosslinking) in all other cases as described in [23].

### Promoter-capture Hi-C (CHi-C)

CHi-C in primary AML cells was performed as described in [4].

### DNase I-seq and ATAC-seq data analysis

Details of the bioinformatics analyses can be found in the Supplementary information.

## Results

### *CEBPA*^*N/C*^ AML forms a distinct epigenetic sub-group as compared to other AML subtypes

In t(8;21) the ETO gene is fused to the gene encoding RUNX1, creating the fusion protein RUNX1/ETO [24]. The expression and open chromatin profiles of *CEBPA*^*N/C*^ and t(8;21) AML clustered closely together [4]. We originally explained this feature with the observation that both mutations affect the same pathway, as *CEBPA* is mutated in *CEBPA*^*N/C*^ AML and is repressed by RUNX1/ETO in t(8;21) AML [24, 25]. To substantiate that *CEBPA*^*N/C*^ represents a specific epigenetic subclass of AML, we repeated these analyses with additional samples to include a total of 10 *CEBPA*^*N/C*^ patients, and studied 7 t(8;21) patients. CD34+ leukemic blasts were purified for RNA-seq and ATAC-or DNase-seq experiments (Supplementary Table S1 and Supplementary Fig. S1A, B). Unsupervised clustering analysis of RNA-seq (Supplementary Fig. S1A) and ATAC/DNase-seq data (Supplementary Fig. S1B) showed that the data from all *CEBPA*^*N/C*^ patients clustered as a unique group. To define the cis-regulatory elements distinguishing *CEBPA*^*N/C*^ from t(8;21) AML, we compared the open chromatin profiles (DNAse-Seq and ATAC-Seq data) of t(8;21) and *CEBPA*^*N/C*^ and also compared both AML subtypes to normal CD34+ peripheral blood stem cells (PBSCs) (Fig.1B and Supplementary Fig. S1C). Individual open chromatin patterns within each subtype were highly similar (Supplementary Fig. S1C, DNase I/ATAC-Seq panel) and thus data could be merged. When compared to each other and to PBSCs, both subtypes displayed numerous specific peaks (Supplementary Fig. S1C). Motif enrichment analysis of specific open chromatin regions revealed that only RUNX and ETS motif enrichment was shared (Fig. 1D, E). Examples of genes and accessible chromatin sites being differentially regulated between the two AML subtypes are shown in Supplementary Fig. S1D, E. Two findings were noteworthy: (i) the occurrence of a CEBP:AP-1 composite motif (Fig.1B–D), which has been shown to bind JUN-CEBP heterodimers [26], and (ii) the enrichment of motifs for POU4F1 (Fig. 1C), a transcription factor that is aberrantly expressed in both *CEBPA*^*N/C*^ and t(8;21) AMLs [4], in the *CEBPA*^*N/C*^-specific peaks.

**Fig. 2:**
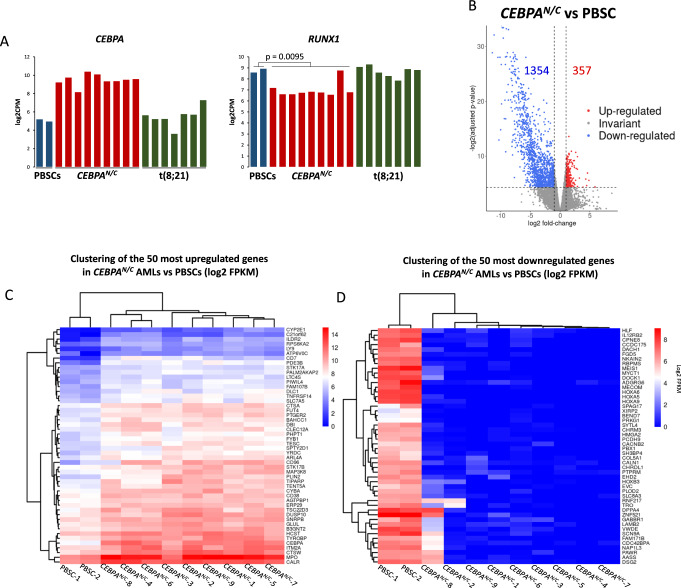
Comparison of primary *CEBPA*^*N/C*^ to normal and t(8;21) AML cells. **A** mRNA expression levels as measured by RNA-seq of *CEBPA* and *RUNX1* in the indicated blast types. *RUNX1* expression: adjusted *P* value 0.0095, Limma–Voom with Benjamini–Hochberg correction for multiple testing. **B** Volcano plot of genes deregulated in *CEBPA*^*N/C*^ AMLs compared to PBSCs. Each dot represents a gene, the log2 fold-change indicates the mean expression level for each gene across all *CEBPA*^*N/C*^ samples. Blue dots represent significantly downregulated genes (log2 fold-change <–1, Benjamini–Hochberg adjusted *P* value <0.05); red dots represent significantly upregulated genes (log2 fold-change >1, Benjamini–Hochberg adjusted *P* value <0.05). **C**, **D** Hierarchical clustering of expression values for the 50 most upregulated (**C**) and downregulated (**D**) genes in *CEBPA*^*N/C*^ AMLs compared to PBSCs.

We next assessed the expression of all *CEBP* genes in both healthy PBSCs, *CEBPA*^*N/C*^ and t(8;21) leukemic blast cells (Fig 2A, left panel, Supplementary Fig. S2A). All were expressed in both subtypes of AML and could potentially compensate for aberrant C/EBPα function. We then compared merged transcriptomic data from *CEBPA*^*N/C*^ AML blasts with healthy PBSCs. In *CEBPA*^*N/C*^ AML blasts, 1354 genes were downregulated and 357 upregulated (Fig. 2B). Patient-specific expression patterns (Supplementary Fig. S1F) and the pattern of highly up- and downregulated genes (Fig. 2C, D) were consistent amongst all 10 patients. The analysis of pathways aberrantly regulated in *CEBPA*^*N/C*^ AML as compared to normal blast cells revealed “ECM-receptor interaction” and “Adherent junctions” as downregulated pathways, in accordance with previous observation that deregulation of stem cell-niche interactions is a hallmark of AML [27] (Supplementary Fig. S2B). Upregulated pathways included “Ribosome” (Supplementary Fig. S2C) indicating increased protein synthesis in highly proliferating leukemic blasts. Note that C/EBPα recruits PolI to stimulate rRNA synthesis in myeloid progenitors [28, 29].

RUNX1 was significantly downregulated in all *CEBPA*^*N/C*^ patients except for a sample harboring a heterozygous *RUNX1* 292delC (Fig. 2A, right panel) carrying a premature stop codon. In contrast, the mRNA level of *CEBPA* was increased (Fig. 2A, left panel). *CEBPA*^*N/C*^ showed more downregulated genes as compared to t(8;21) (Supplementary Figs. S2D–F and S3A, B) but the comparison to PBSCs showed that most downregulated genes were shared between the two subtypes (Supplementary Fig. S2G) in contrast to upregulated genes (Supplementary Fig. S2H). This result suggests that the similarities between the two subtypes may be caused by the impediment of C/EBPα function.

### Mutant C/EBPα proteins colocalize with AP-1 and RUNX1 factors in human *CEBPA*^*N/C*^ cells

**Fig. 3:**
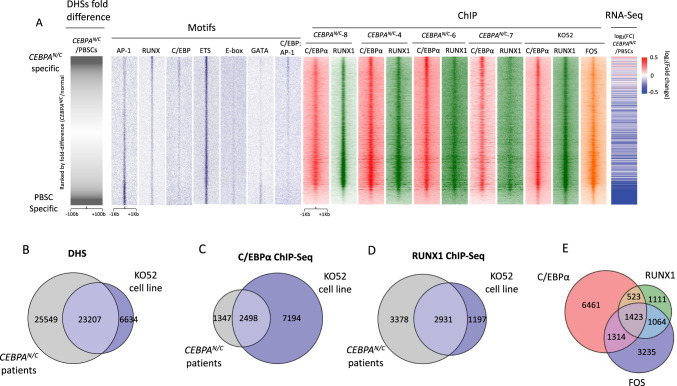
The binding pattern of C/EBPα, RUNX1 and FOS in primary cells and a *CEBPA*^*N/C*^ cell line. **A** Density plot of C/EBPα (red), RUNX1 (green) and FOS (orange) ChIP-Seq. peaks in *CEBPA*^*N/C*^ samples and the KO52 cell line across a 2 kb window. ChIP data, together with TF binding motifs (blue plots) and gene expression data (rightmost plot), are plotted alongside hypersensitive sites ranked by normalized tag counts of merged *CEBPA*^*N/C*^ peaks over merged PBSCs peaks. **B** Venn diagram showing the number of hypersensitive sites shared between the KO52 cell line and *CEBPA*^*N/C*^ patient samples. **C**, **D** Venn diagrams showing the number of C/EBPα (**C**) and RUNX1 (**D**) ChIP peaks shared between KO52 cell line and *CEBPA*^*N/C*^ patient samples. **E** Venn diagram showing the overlap of C/EBPα, RUNX1 and c-FOS ChIP peaks in KO52 cell line.

*CEBPA*^*N/C*^ AMLs express only one DNA-binding form of C/EBPα (p30) (Fig. 1A). To identify genome-wide p30 C/EBPα binding sites, we performed ChIP-seq assays in purified blast cells of four *CEBPA*^*N/C*^ patients. Data were ranked alongside the fold-change between *CEBPA*^*N/C*^ ATAC-Seq peaks and those of healthy PBSCs. We found that the mutant C/EBPα binding patterns were comparable in all samples and were skewed toward *CEBPA*^*N/C*^ patient-specific accessible chromatin sites (Fig. 3A, ChIP panels). RUNX1 and C/EBPα cooperate to regulate myeloid-specific genes [30, 31]. We therefore measured RUNX1 binding and found that binding patterns in all four patients closely followed those of C/EBPα (Fig. 3A, ChIP panels). Motif alignment analysis (Fig. 3A, Motif panels) showed that C/EBPα ChIP peaks were enriched for C/EBP motifs and again showed an enrichment for C/EBP:AP-1 composite motifs only in *CEBPA*^*N/C*^ patient samples. Mutant C/EBPα binding was associated with active and inactive genes (Fig. 3A, outermost panel).

To be able to perform biochemical experiments, we employed the *CEBPA*^*C/N*^ AML cell line model KO52 [15, 32]. A total of 77.8% of all KO52 peaks overlapped with those from patients, demonstrating that the subtype identity of these cells is largely preserved (Fig. 3B). ChIP experiments for C/EBPα, RUNX1 and the AP-1 family member FOS showed that the pattern of binding resembled that of patients (Fig. 3A, right ChIP panel, Supplementary Fig. S3C–E for motif analyses). RUNX1 and C/EBPα ChIP-Seq peaks of patients were mostly contained within the KO52 peak population (Fig. 3C, D) with around 40% of FOS (AP-1) binding sites colocalizing with C/EBPα (Fig. 3E). The majority of RUNX1 peaks overlapped with either C/EBPα (47%) or AP-1 (60%) (Fig. 3E). *RUNX1* itself was bound by all three factors sharing several binding sites (Supplementary Fig. S3F). Bootstrapping analysis, which measures the frequency of TF binding motifs colocalized within 50 bp, confirmed this result (Supplementary Fig. S3G). A detailed analysis of motif spacing revealed a specific arrangement of binding sites, with motifs for AP-1 and C/EBP as well as AP-1 and RUNX1, but not C/EBP and RUNX1 being periodically arranged with a 20 bp distance (Supplementary Fig. S3H). These arrangements were specific for ATAC sites harboring ChIP peaks (Supplementary Fig. S3I, J). In the C/EBPα/AP-1 ChIP sites we found several colocalized binding motifs, most of which turned out to be C/EBP:AP-1 sites, again suggesting factor cooperation. Integration of differentially expressed genes with ChIP data showed that in between 30 and 50% of genes upregulated in *CEBPA*^*N/C*^ AML compared to normal cells were bound by each of the three factors (Supplementary Fig. S4A, B) but this was true only for an average of 25% of downregulated genes (Supplementary Fig. S4C, D), suggesting that this cooperation is important for the establishment of AML-specific gene activity.

### The identification of a core gene regulatory network for CEBPA^N/C^ AML

**Fig. 4:**
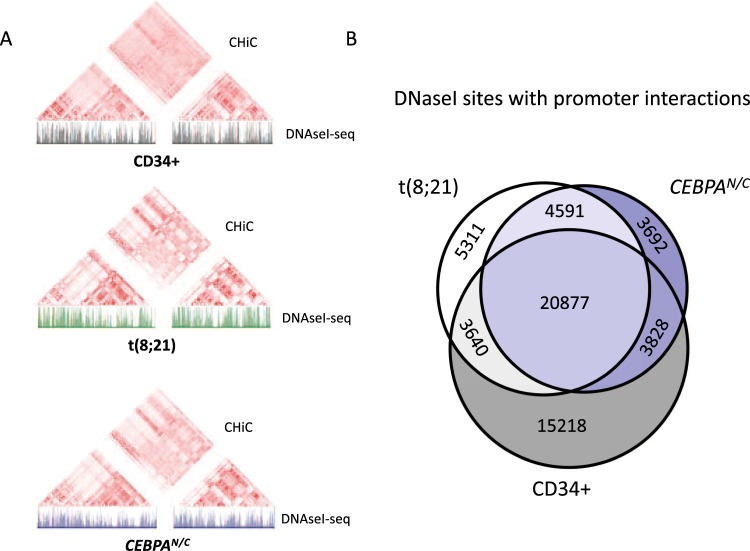
Determination of the nuclear interactome of primary *CEBPA*^*N/C*^ blasts. **A** Contact matrix across chromosome 1 for healthy CD34^+^ blasts (top) and t(8;21) (middle) and *CEBPA*^*N/C*^ (bottom) leukemic blasts at 10 Mb resolution. A UCSC genome browser track is shown below each matrix to highlight the DNAse I hypersensitive site pattern. **B** Overlap of DNase I-HS sites with interactions for t(8;21) and *CEBPA*^*N/C*^ AML as compared to normal CD34+ cells.

The definition of the GRN on which *CEBPA*^*N/C*^ AMLs rely requires the assignment of regulatory sequences to their target promoters. We therefore defined the whole set of distal element-promoter interactions for one of our *CEBPA*^*N/C*^ patient samples (*CEBPA*^*N/C*^*-9*, Supplementary Table S1) using promoter-capture Hi-C (CHi-C). The interactome for both normal CD34+ cells and t(8;21) patients was previously characterized [4, 33]. As expected, most interactions occurred within the same chromosome (Supplementary Fig. S4E) and the organization into TADs in PBSCs, *CEBPA*^*N/C*^ and t(8;21) AMLs did not differ (Fig. 4A). However, when zooming in on individual genes, differences became apparent, as shown with the *SPI1* locus (Supplementary Fig. S4F). More than 100,000 interactions between distal open chromatin regions and promoters were identified in each sample, and ~88% of *CEBPA*^*N/C*^ interactions were also present in the t(8;21) dataset (Supplementary Fig. S5A). A similar overlap was also seen when compared to healthy CD34+ cells. To gain further insight into the biology of *CEBPA*^*N/C*^ AML we identified the cis-regulatory elements underlying *CEBPA*^*N/C*^ specific interactions by assigning them to open chromatin regions. 3692 DHSs were within this population (Fig. 4B). We assigned those elements to their rightful genes using CHi-C (Supplementary Dataset 1) and compared gene expression to PBSCs (Supplementary Table S2 and Supplementary Dataset 2). *CEBPA*^*N/C*^ specific DHSs were mostly associated with downregulated genes important for myelopoiesis, including *MAF* and *RUNX1*. 72.5% of upregulated and 61.9% of downregulated genes were C/EBPα targets, demonstrating a major role of the oncogene in shaping the AML subtype-specific chromatin landscape.

**Fig. 5:**
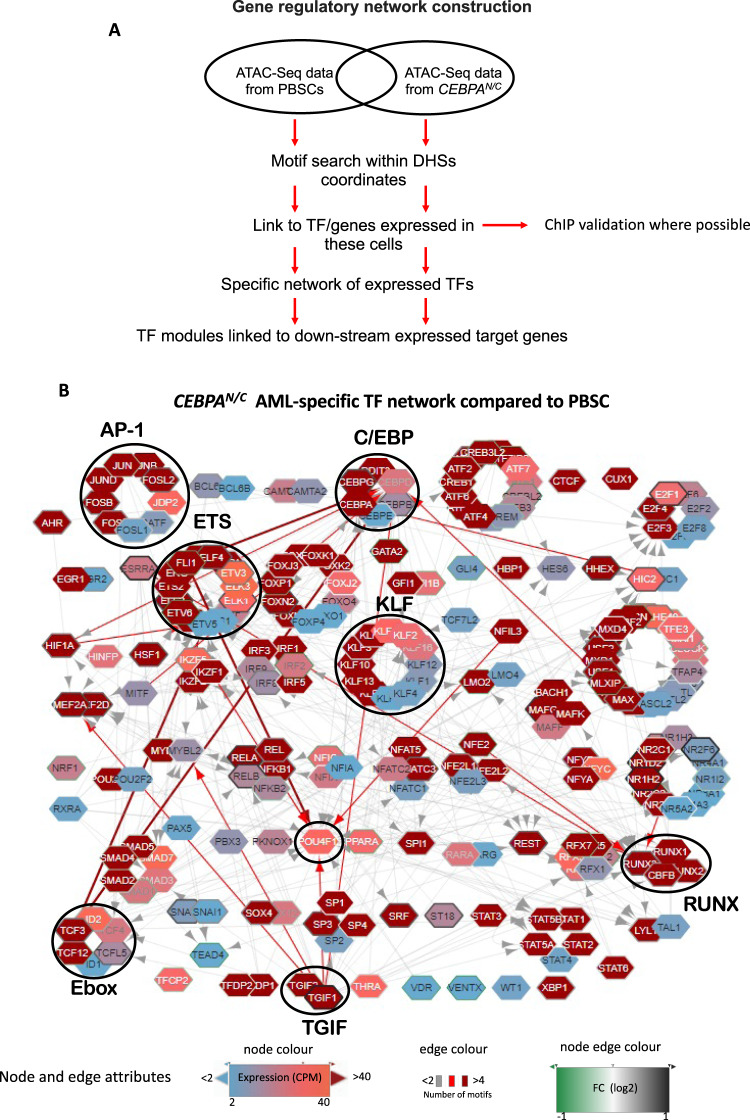
The core transcription factor network of primary *CEBPA*^*N/C*^ cells. **A** Network construction strategy overview based on the aggregate of data from 10 patients. **B** TF targets determined by known motifs as described in [4]. Arrows (Edges) pointing outward from entire node highlight motifs present in individual genes whereby the hypersensitive site was annotated to the gene using the CHi-C data where possible, or otherwise to the nearest gene. The expression level of the individual genes (CPM) in *CEBPA*^*N/C*^ samples is depicted in blue (low) or red (high) color. Edges are colored accordingly with the number of motifs. Node edge color indicates the gene expression level in *CEBPA*^*N/C*^ samples compared to PBSCs.

To construct the *CEBPA*^*N/C*^-specific GRN, we first defined the potential cis-regulatory elements deregulated in leukemic blasts as compared to PBSCs (Fig. 5A) [4]. We then performed a motif search within these DHSs to infer candidate TFs that bind these sites by using a list of non-redundant motifs representing 80 TF families expressed in myeloid cells [4]. Using CHi-C data, we linked each binding motifs to the promoters of the genes that they regulate (Fig. 5B). Each network node represents a TF colored according to its expression in *CEBPA*^*N/C*^ AMLs. TF families binding to the same motif form a composite node (encircled), arrows going from a node to a specific gene indicate the presence of a binding motif in the locus encoding that gene. The analysis highlights nodes and edges which are specific for *CEBPA*^*N/C*^ AMLs and how different TFs and their genes are “wired”. The C/EBP and AP-1 families represent major nodes, with 341 and 353 connections to putative targets, respectively (Supplementary Dataset 3), suggesting a high regulatory relevance. ETS, RUNX and E-box factors, which are part of the global hematopoietic signature [34], are highly connected, with the RUNX family being linked to about 500 genes (Supplementary Dataset 3).

Our GRN construction strategy relies on the identification of TF binding motifs. For validation, we compared C/EBP, RUNX and AP-1 targets identified in the *CEBPA*^*N/C*^-specific GRNs with C/EBPα, RUNX1 and c-FOS ChIP data. Almost 50% of AP-1 family targets identified in our GRNs were bound by c-FOS in KO52 cells (Supplementary Fig. S5B). The overlap was over 60% for the RUNX1 (Supplementary Fig. S5C) and over 80% for the C/EBP targets (Supplementary Fig. S5D), demonstrating the reliability of our strategy. The comparison of AML subtype-specific pathways from the three modules showed little overlap, with the most deregulated pathways in the AP-1 module being associated with signaling and cell cycle as expected (Supplementary Fig. S5B–D and Supplementary Dataset 4).

### Analysis of *CEBPA*^*C/N*^ leukemic stem cells (LSCs) and blast populations at the single cell level

**Fig. 6:**
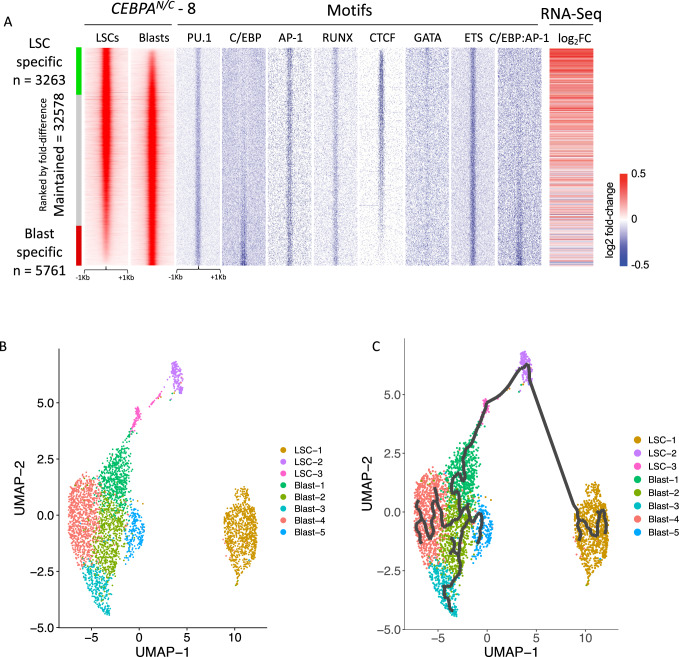
Chromatin differences between *CEBPA*^*N/C*^ leukemic stem and blast cells. **A** ATAC-seq peak profile comparison of LSC and blast cells purified from *CEBPA*^*N/C*^*-8* patient sample across a 2 kb window. Data are ranked by normalized tag counts of LSC peaks over blasts. TF binding motifs projected against hypersensitive sites and fold differences in gene expression associated with open chromatin regions are plotted alongside. **B** Analysis of scRNA-Seq data. Uniform Manifold Approximation and Projection for Dimensional Reduction (UMAP) map displaying LSC and blast clusters as identified with GSEA analysis. Each dot in the map represents a cell and is colored accordingly to cluster assignment. **C** Monocle pseudotime trajectory of LSC and blast cells projected on the UMAP map of scRNA clusters.

LSCs are a rare subpopulation among leukemic blasts that survives chemotherapy [35]. To identify LSC-specific vulnerabilities, we characterized LSCs (CD34+ CD90– CD38–) and leukemic blasts (CD34+ CD90– CD38+) purified from a *CEBPA*^*N/C*^ AML sample expressing both C/EBPα isoforms (Supplementary Fig. S6A–C). ATAC-seq analysis showed 3263 open chromatin sites enriched in LSCs which were associated with specific gene expression program, and 5761 enriched in blasts, (Fig. 6A, Supplementary Fig. 6D and Supplementary Dataset 5). The analysis of the gene expression profile showed an LSC score [36] of –0.22 in LSC populations compared to –0.45 in blasts, confirming the nature these cells as LSCs (Supplementary Fig. S6E). Motif analysis showed enrichment of C/EBP motifs in blast-specific sites while GATA motifs were more represented in LSC-specific sites, in accordance with their grade of differentiation (Fig. 6A). The composite C/EBP:AP-1 motif was specifically enriched in the blast-specific ATAC-Seq sites (Fig. 6A).

Bulk ATAC-seq analysis highlights differences in the LSC and blast motif signatures but does not take into account that each of these populations represents a continuum of differentiation stages. We thus performed single cell (sc)RNA-seq on LSCs and blasts from one *CEBPA*^*N/C*^ patient sample. Clustering analysis revealed 8 cell clusters (Fig. 6B). We then compared the sets of marker genes from each cluster to an LSC/Blast gene expression signature derived from our bulk RNA-Seq data using GSEA, which identified three clusters as LSC, with the remaining five identified as blasts (Supplementary Fig. S6F, G). The analysis of genes expressed in different phases of the cell cycle showed that LSCs were mainly in G0/G1 phase, consistent with their quiescent state. The cell cycle status of blast cells was more variable, with the Blast-5 cluster showing the highest proliferation rate (Supplementary Fig. S6H, I). In a pseudotime analysis, the LSC-1 cluster was localized at the apex of the differentiation trajectory (Fig. 6C) with the strong enrichment of a LSC gene expression signature (Supplementary Fig. S6G). The Blast-1 population gives rise to Blast-2, which subsequently branches into other three blast populations (Fig. 6C and Supplementary Fig. S6I).

**Fig. 7:**
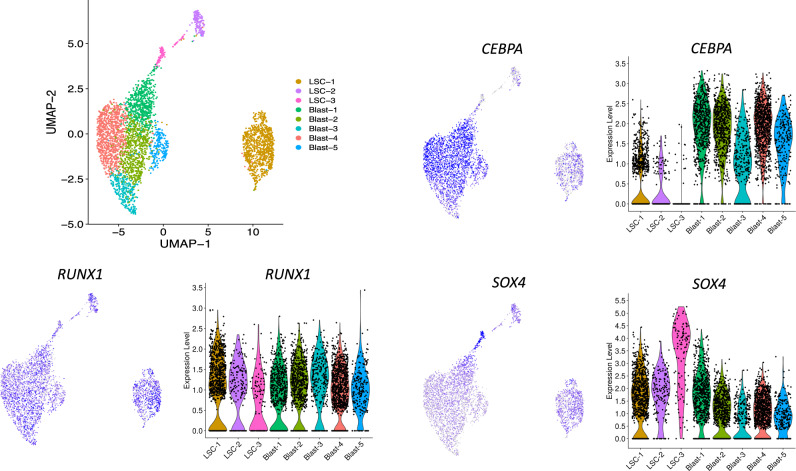
Cell stage-specific expression of different regulator genes in *CEBPA*^*N/C*^ LSCs and blast cells. Expression of indicated genes projected on the UMAP map (upper left panel). Color intensity represent expression data log2 normalized unique molecular identifier (UMI) counts.

We then projected the expression of genes encoding important hematopoietic TFs that form nodes in the *CEBPA*^*N/C*^ AML GRN on the cell clusters (Fig. 7 and Supplementary Fig. S7). As expected, *CEBPA* expression was increased in all blast clusters compared to LSCs. *SOX4* was expressed at higher levels in the LSC compartment (Fig. 7, for statistical significance see Supplementary Fig. S8A). In this context, note that *SOX4* is repressed by wild-type C/EBPα and is required for self-renewal in *CEBPA*^*N/C*^ LSCs [37]. The aberrantly expressed TF POU4F1 is expressed only within the LSC-1 cluster (Supplementary Fig. S7). The AP-1 family member FOS is expressed across all clusters, while its partner JUN is preferentially expressed in LSCs (Supplementary Fig. S7).

### The growth of *CEBPA*^*N/C*^ AML is dependent on RUNX1 as well as AP-1, and C/EBP TF families

C/EBP, RUNX and AP-1 families constitute prominent nodes of the *CEBPA*^*N/C*^-specific GRN. To investigate to which extent these TFs sustain the aberrant leukemic program, we generated lentiviral constructs expressing doxycycline(Dox)-inducible dominant negative C/EBP (dnC/EBP) [20] and FOS (dnFOS) [38] peptides, which heterodimerize with multiple members of the C/EBP and AP-1 families, respectively, and abolish their DNA binding, or a GFP-expressing empty vector control [21]. We could not transduce dnC/EBP and dnFOS vectors into *CEBPA*^*N/C*^ patient blasts and therefore used KO52 and the t(8;21) cell line Kasumi-1 (Supplementary Figs. S8B and S9A–D).

**Fig. 8:**
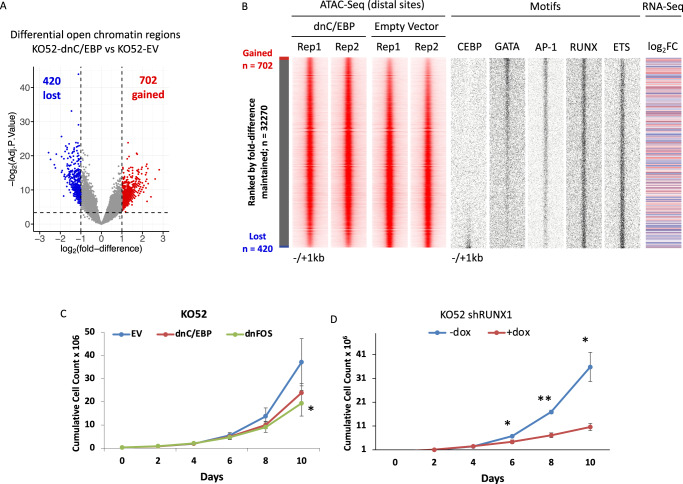
Expression of dominant versions of C/EBP and FOS is detrimental for *CEBPA*^*N/C*^ cell survival. **A** Volcano plot of hypersensitive sites deregulated in KO52 cells expressing dnC/EBP compared to control. Blue and red dots represent significantly downregulated (log2 fold-change <–1, Benjamini–Hochberg adjusted *P* value <0.05) and significantly upregulated (log2 fold-change >1, Benjamini–Hochberg adjusted *P* value <0.05) hypersensitive sites. Right panel: motif enriched in dnC/EBP-specific and control-specific hypersensitive sites. **B** Density plots showing the ATAC-seq profile of KO52 cells expressing dnC/EBP or a control vector across a 2 kb window (2 replicates). Data are ranked by normalized tag counts of dnC/EBP expressing cells over control. TF binding motifs projected against hypersensitive sites are plotted alongside. **C** Cumulative growth time course of KO52 cells expressing either a control vector, dnC/EBP or dnFOS (*N* = 3). **D** Cumulative growth time course of KO52 cells expressing an inducible shRNA targeting *RUNX1* with or without doxycycline (*N* = 3). In **C** and **D**, error bars represent the sample standard deviation. *P* values were calculated using a two-tailed *t*-test.

After Dox treatment and FACS sorting for GFP positive cells, we performed ATAC-seq and RNA-seq on transduced cells. After induction of dnC/EBP expression for 72 h in KO52 cells, ATAC analyses identified 420 lost and 702 gained open chromatin regions (Fig. 8B). Gained ATAC sites were enriched in AP-1, RUNX1, ETS and GATA motifs, whereas lost sites were enriched in PU.1/ETS, RUNX and C/EBP motifs (Fig. 8B and Supplementary Fig. S8C), suggesting that C/EBP binding was lost. However, we observed minimal changes in gene expression (Supplementary Fig. S8D) with 31.1% of upregulated and 47.4% of downregulated genes being C/EBPα targets (Supplementary Fig. S8E). ATAC-seq experiments in Kasumi-1 cells after inducing dnC/EBP revealed that 805 open chromatin regions enriched in C/EBP motifs were lost (Supplementary Fig. S9A–C), demonstrating a higher sensitivity of this AML subtype against C/EBP inhibition.

The induction of dnFOS in KO52 cells resulted in a gain of 191 open chromatin regions, which were enriched in PU.1 and ETS motifs, and loss of 857 sites with a significant enrichment of RUNX, GATA and AP-1 motifs (Supplementary Fig. S10A–C), suggesting that closing of chromatin was due to loss of AP-1 binding. Gene expression analysis of dnFOS transduced cells showed 288 differentially regulated genes, with most genes (180) being downregulated (Supplementary Fig. S10D). KEGG pathway analysis showed that various downregulated genes encode for cell signaling components, such as SRC and multiple chemokines (Supplementary Fig. S10E), together with *CEBPE* which is bound by FOS, C/EBPα and RUNX1 (Supplementary Fig. S10F).

To test whether C/EBPs and AP-1 family members were required for leukemic cell growth, we performed proliferation assays with KO52 and Kasumi-1 cells expressing dominant negative constructs. Both dnC/EBP and dnFOS expression decreased cell growth in our *CEBPA*^*N/C*^ and t(8;21) cell line models ((Fig. 8C and Supplementary Fig. S9D) and [4]). Using a DOX-inducible shRNA (Supplementary Fig. S11B), we also confirmed that JUND is required for efficient proliferation of KO52 cells (Supplementary Figs. S10G and S11A). The presence of dnC/EBP and dnFOS constructs in KO52 cells led to an increased loss of GFP+ cells as compared to the induced empty vector construct (Supplementary Fig. S11B), suggesting that the expression of the peptides is toxic.

RUNX1 has been shown to be essential for the survival of t(8;21) AML [39]. We therefore depleted RUNX1 directly using a DOX-inducible shRNA (Fig. 8D and Supplementary Fig. 11C). We also employed a small molecule inhibitor of CBFβ (AI-14-91), which disrupts its interaction with the RUNT domain of RUNX proteins and has been shown to affect the survival of AML but not normal cells [23, 40] (Supplementary Fig. S10H). These experiments demonstrate that the RUNX1 inhibitor, but not the control compound AI-4-88, and RUNX1 depletion strongly reduce KO52 growth, with an increase in apoptosis (Supplementary Fig. S11D). FACS analyses demonstrated a modest change in the differentiation state of the cells, with JUND depletion increasing and RUNX1 depletion decreasing CD34+ cell numbers (Supplementary Fig. S11E–H), suggesting that the balance of RUNX1, AP-1 and mutant C/EBP impacts on the differentiation state of the cells. Taken together, these data show that our GRN analysis is predictive, demonstrating that the highly connected network nodes RUNX1, AP-1 and C/EBP are essential for regulating the growth of *CEBPA*^*N/C*^ and t(8;21) cells.

## Discussion

### AP-1 and C/EBP shape the regulatory phenotype of CEBPA^N/C^ AMLs

C/EBPα is required for myeloid differentiation and for maintaining the self-renew ability and the quiescent state of adult HSCs [41, 42]. Therefore, it was important to examine how and where mutant proteins interact with the genome and regulate gene expression. Our data suggest that mutant C/EBPα proteins cooperate with other TFs, as indicated by the presence of the composite C/EPB:AP-1 motif in *CEBPA*^*N/C*^-specific DHSs and the colocalization of C/EBPα, RUNX1 and AP-1, in contrast to the enrichment of the AP-1 canonical motif in t(8;21) and healthy blasts-specific sites. We showed previously that AP-1 activity is required for leukemic maintenance in FLT3-ITD and t(8;21) AMLs [4, 21, 43]. Here we show that this is also true for *CEBPA*^*N/C*^. C/EBP and AP-1 TFs form heterodimers [44–46], and C/EBPα:AP-1 interaction is required to drive monocytic lineage commitment [26]. Our data indeed suggest such collaboration. C/EBP and the C/EBP:AP-1 motif signatures were mainly confined to blast cells, suggesting that cooperation may be involved in maintaining this specific differentiation state. Our single cell experiments confirmed these differences. Except for *JUN*, AP-1 family members were uniformly expressed throughout all LSC and blast subpopulations. *JUN* was downregulated in the blast compartment, following an expression pattern inversely correlated with *CEBPA*. During granulocyte differentiation C/EBPα heterodimerizes with JUN, impeding the formation of JUN:FOS complexes and preventing JUN from autoregulate its own promoter. Therefore, JUN downregulation promotes granulocytic differentiation [47].

The *CEBPA*^*N/C*^-specific core GRN Highlights the AML-specific connections between TF-coding genes that are distinct from those found in PBSCs. The analysis of the TF modules (Supplementary Dataset 3), representing the downstream targets of TFs forming nodes, shows that these differences are responsible for the alterations in gene expression in AML.

Another important result described here is the finding that despite one essential member being mutated, the C/EBP family represents a highly connected node of the *CEBPA*^*N/C*^ specific GRN and thus contributes to shaping the regulatory landscape. Note that a better clinical outcome of *CEBPA* mutant AML is associated with cells containing C/EBPα bZIP domain mutation, irrespective of whether it is co-expressed with a wt protein or a C-terminal mutant as in *CEBPA*^*N/C*^ AML [11]. The bZIP mutant cannot bind/dimerize but could still be part of the complex and thus could interfere with multiple C/EBP proteins. Whether both mutant proteins are associated with chromatin is unclear since our antibody cannot distinguish between them. However, our colocalization studies point to an association of mutant C/EBPα proteins with other TFs which could allow some myeloid differentiation, thus improving prognosis. This finding is entirely consistent with the fact that the *CEBPA*^*N/C*^ GRN (i) contains C/EBP proteins as a major node, (ii) that a large number of genes are bound by the mutant protein, including *C/EBPA*^*N/C*^-AML-specific cis-elements, (iii) that the latter are associated with downregulated myeloid differentiation genes and (iv) that eliminating this node using dnC/EBP affects the survival of the cells.

Within the *CEBPA*^*N/C*^ GRN, the RUNX TF family is highly connected. Targets of RUNX1 in the AML-specific GRN include, for example, the apoptosis regulator *BCL2* which is upregulated in *CEBPA*^*N/C*^ AML blasts, and which could be inhibited by Venetoclax [48]. Similar to other AML subtypes [4], the AP-1 family forms an important node in the *CEBPA*^*N/C*^ AML GRN, indicating that MAPK signaling plays an essential role in regulating growth. For FLT3-ITD and t(8;21) AML this idea has been confirmed in vitro and in vivo [4, 21].

Inhibition of the RUNX TF family and the depletion of RUNX1 shows that it is important for *CEBPA*^*N/C*^ AML growth. Expression of dnFOS had a strong effect on the chromatin landscape of KO52 cell, mainly causing loss of open chromatin sites. Interestingly, C/EBP:AP-1 composite motifs were enriched in hypersensitive sites lost upon dnC/EBP expression in KO52, but not in Kasumi-1 cells. This result suggests that the cooperation between these TFs is a specific feature of *CEBPA*^*N/C*^ AMLs. Our gene expression analysis showed that loss of AP-1 binding in KO52 was accompanied by downregulation of signaling pathways, suggesting that these factors promote the survival of *CEBPA*^*N/C*^ cells. Finally, we found that the expression of dnC/EBP or dnFOS induced growth arrest in both KO52 (this study) and Kasumi-1 cells (this study and [23]).

In summary, the *CEBPA*^*N/C*^ AML-specific core GRN highlights subtype-specific connections between TFs and target genes that code for “druggable” gene products. It will be an important resource for researchers developing combination therapies for this particular AML subtype.

## Supplementary information

Supplementary Material

Supplementary Dataset 1

Supplementary Dataset 2

Supplementary Dataset 3

Supplementary Dataset 4

Supplementary Dataset 5

## Data Availability

Data can be accessed at NCBI GEO (https://www.ncbi.nlm.nih.gov/geo/) under GSE211095.
