# Supplementary material for: Identification and interrogation of the gene regulatory network of CEBPA-double mutant acute myeloid leukemia
Source: Leukemia. 2022 Nov 4;37(1):102–12. doi: 10.1038/s41375-022-01744-5 (PMC9883165; doi:10.1038/s41375-022-01744-5)
Supplement: Supplementary file 1 — Supplementary Material [file 41375_2022_1744_MOESM1_ESM.pdf]

Identification and interrogation of the gene regulatory network of CEBPA-double mutant Acute Myeloid Leukaemia" by Adamo, Chin et al.

List of Supplementary Information

1. **Supplementary Tables, Figures and Figure Legends**
2. **Supplementary Methods and references**
3. **Supplementary Datasets**

S1: Chromosome position of open chromatin regions and their associated genes linked by CHiC

S2: RUNX, CEBP and FOS GRN modules with gene expression data and ChIP target status

S3: KEGG pathways associated with CEBP$^{N/C}$-specific AP-1, C/EBP and RUNX modules

S4: Bulk RNA-Seq analysis of sorted LSC vs. Blast populations

**Patient code**	**t(8;21)**	**CEBPA**	**Other mutations**
PBSC-1			
PBSC-2			
CEBPA$^{N/C}$-1		*CEBPA*$^{N/C}$	
CEBPA$^{N/C}$-2		*CEBPA*$^{N/C}$	GATA2
CEBPA$^{N/C}$-3		*CEBPA*$^{N/C}$	GATA2, TET2
CEBPA$^{N/C}$-4		*CEBPA*$^{N/C}$	FLT3-ITD
CEBPA$^{N/C}$-5		*CEBPA*$^{N/C}$	GFI1_SNP
CEBPA$^{N/C}$-6		*CEBPA*$^{N/C}$	DNMT3A
CEBPA$^{N/C}$-7		*CEBPA*$^{N/C}$	
CEBPA$^{N/C}$-8		*CEBPA*$^{N/C}$	RUNX1, TET2, JAK2
CEBPA$^{N/C}$-9		*CEBPA*$^{N/C}$	del9q
CEBPA$^{N/C}$-10		*CEBPA*$^{N/C}$	DNMT3A, del(11)(q14 q25)
t(8;21)-1	t(8;21)		TET2
t(8;21)-2	t(8;21)		TET2, KIT
t(8;21)-3	t(8;21)		FLT3-TK
t(8;21)-4	t(8;21)		NOTCH1, KIT
t(8;21)-5	t(8;21)		
t(8;21)-6	t(8;21)		FLT3-ITD
t(8;21)-7	t(8;21)		
inv(16)-1			inv(16), KIT
inv(16)-2			inv(16)
inv(16)-3			inv(16), ASXL1
inv(3)/RAS-1			inv(3), GATA2, SF3B1, NRAS
inv(3)/CBL-2			inv(3), SF3B1, CBL
ITD-1			DNMT3A, TET2x2, BCOR, TP53, FLT3-ITD
ITD-2			DNMT3A, TET2, FLT3-ITD, tri(13)
ITD-3			DNMT3A, FLT3-ITD
ITD(2x)/NPM1-1			DNMT3A, IDH2, NPM1, FLT3-ITDx2
ITD(2x)/NPM1-2		CEBPA	IDH2, NPM1, FLT3-ITDx2
ITD/NPM1-1			WT1, DNMT3A, NPM1, FLT3-ITD
ITD/NPM1-2			NPM1, FLT3-ITD
ITD/NPM1-3			NPM1, FLT3-ITD
ITD/NPM1-4			GATA2, DNMT3A, NPM1, FLT3-ITD
ITD/NPM1-5			DNMT3A, BCOR, NPM1, FLT3-ITD
ITD/NPM1-6			WT1, DNMT3A, TET2, PHF6, NPM1, FLT3-ITD
NPM1-1			IDH1, NPM1
NPM1-2			DNMT3A, TET2x2, NPM1
NPM1/RAS-3			PTPN11, DNMT3A, IDH1, NPM1, NRAS
RUNX1-DT-1			CREBBP, DNMT3A, SF3B1, RUNX1, FLT3, tri(13)
RUNX1-DT/CEBPA-2		CEBPA	WT1x2, SF3B1, TP53, RUNX1, FLT3-ITD
RUNX1-DT-3			RUNX1
RUNX1(x2)-D&T-4			DNMT3A, IDH2, SRSF2, RUNX1x2
RUNX1-D-5			IDH1, BCORL1x2, SRSF2x2, RUNX1
RUNX1-T/CEBPA-6		CEBPA	EZH2, RUNX1, NRAS, tri(8)
RUNX1-T-7 (NHL)			TET2x2, PHF6, RUNX1, tri(21)

Supplementary Table 1: Patient mutation data. Patient samples (were obtained from either the Haematological Malignancy Diagnostic Service (HMDS) in Leeds, the University Hospital Birmingham NHS trust and from the sample collection of the Erasmus MC Cancer Institute, Rotterdam.

Significantly enriched GO terms for genes that are Up-Regulated and have a *CEBPA*$^{N/C}$ specific interaction					
**ID**	**Term**	**No of genes**	**p.value**	**adj. p.value**	**Genes**
GO:0010155	regulation of proton transport	3	1.8E-05	5.4E-05	PPIF, TESC, TMSB4X
GO:0032272	negative regulation of protein polymerization	3	8.4E-04	1.3E-03	CAPG, TMSB4X, VDAC2
GO:0042274	ribosomal small subunit biogenesis	3	1.1E-03	1.1E-03	NPM1, RPS21, RPS24

Significantly enriched GO terms for genes that are Down-Regulated and have a *CEBPA*$^{N/C}$ specific interaction					
**ID**	**Term**	**No of genes**	**p.value**	**adj. p.value**	**Genes**
GO:0002062	chondrocyte differentiation	5	6.8E-04	3.8E-03	MAF, RUNX1, SERPINH1, SMAD3, TRPS1
GO:0007368	determination of left/right symmetry	5	1.7E-03	5.2E-03	MICAL2, PCSK5, RFX3, SMAD3, VANGL2
GO:0045638	negative regulation of myeloid cell differentiation	5	3.2E-04	9.0E-03	FBN1, MEIS1, RUNX1, TJP2, TMEM178A
GO:0061515	myeloid cell development	4	1.2E-03	4.6E-03	FBN1, MEIS1, TJP2, TSPAN2
GO:0030500	regulation of bone mineralization	4	1.4E-03	4.8E-03	ANKH, BMP2K, SLC8A1, SMAD3
GO:1902106	negative regulation of leukocyte differentiation	4	4.6E-03	7.2E-03	FBN1, RUNX1, TJP2, TMEM178A
GO:0043506	regulation of JUN kinase activity	4	3.5E-03	6.5E-03	DTNBP1, TIAM1, TNIK, VANGL2
GO:0002762	negative regulation of myeloid leukocyte differentiation	4	4.9E-04	4.6E-03	FBN1, RUNX1, TJP2, TMEM178A
GO:0003044	regulation of systemic arterial blood pressure mediated by a chemical signal	4	5.3E-04	3.7E-03	ADRB1, ECE1, PCSK5, TPM1
GO:0072384	organelle transport along microtubule	4	2.1E-03	5.2E-03	ARHGAP21, DTNBP1, RASGRP1, SUN1
GO:0070167	regulation of biomineral tissue development	4	2.4E-03	5.5E-03	ANKH, BMP2K, SLC8A1, SMAD3
GO:0010717	regulation of epithelial to mesenchymal transition	4	2.6E-03	5.6E-03	PHLDB1, SMAD3, TCF7L2, TIAM1
GO:0048640	negative regulation of developmental growth	4	3.4E-03	6.7E-03	ADRB1, BCL11A, MEIS1, SEMA4F
GO:0045671	negative regulation of osteoclast differentiation	3	9.9E-04	4.6E-03	FBN1, TJP2, TMEM178A
GO:0045824	negative regulation of innate immune response	3	5.6E-03	8.2E-03	SERPINB9, SERPING1, TYRO3
GO:0001779	natural killer cell differentiation	3	4.2E-04	5.8E-03	IL15, RASGRP1, TYRO3
GO:0098751	bone cell development	3	1.8E-03	4.9E-03	FBN1, MEIS1, TJP2
GO:0032330	regulation of chondrocyte differentiation	3	4.4E-03	7.3E-03	MAF, SMAD3, TRPS1
GO:0045010	actin nucleation	3	5.6E-03	8.2E-03	ACTR3C, IQGAP2, SPIRE1
GO:0010718	positive regulation of epithelial to mesenchymal transition	3	3.7E-03	6.5E-03	SMAD3, TCF7L2, TIAM1
GO:0008347	glial cell migration	3	5.6E-03	8.2E-03	SUN1, TIAM1, VCAN
GO:0002448	mast cell mediated immunity	3	5.9E-03	8.3E-03	RASGRP1, SERPINB9, SLC7A8

Supplementary Table 2: Gene ontology terms for genes associated with DHS with *CEBPA*$^{N/C}$ specific interactions which are up-regulated (upper panel) or down-regulated (lower panel) compared to PBSCs.

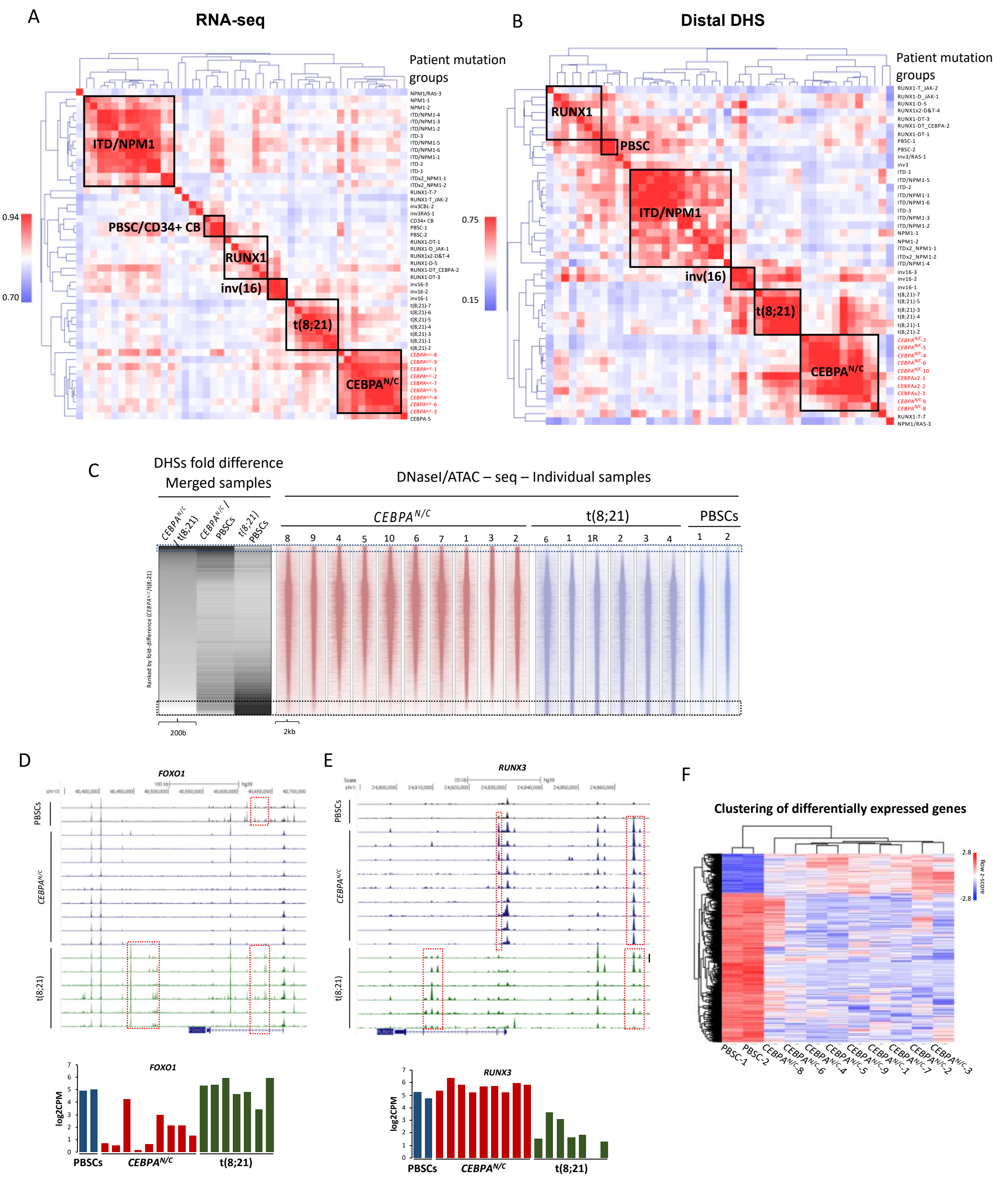

Figure S1: Comparison of open chromatin regions in blasts of patients with *CEBPA*$^{N/C}$, t(8;21) and healthy PBSCs (A) and (B) Hierarchical clustering of Pearson correlation coefficients of RNA-seq (A) and DNase I/ATAC-seq (B) data from(4) and this study. *CEBPA*$^{N/C}$ patient samples are highlighted in red. (C) Leftmost plot: Hypersensitive sites fold difference between *CEBPA*$^{N/C}$ and t(8;21), *CEBPA*$^{N/C}$ and PBSCs and t(8;21) and PBSCs samples respectively, across a 200 bp window. Rightmost plots: Density plots showing the open

chromatin profile of *CEBPA*$^{N/C}$ (pink), t(8;21) (lilac) and PBSC (pale blue) across a 2kb window. Data are ranked by normalized tag counts of merged *CEBPA*$^{N/C}$ peaks over merged PBSCs peaks. (D-E) Gene expression and chromatin accessibility patterns at the indicated loci. (F) Hierarchical clustering heatmap of RNA-seq expression z-scores computed for genes differentially expressed between *CEBPA*$^{N/C}$ and PBSCs samples. Each row represents a gene.

Figure S2

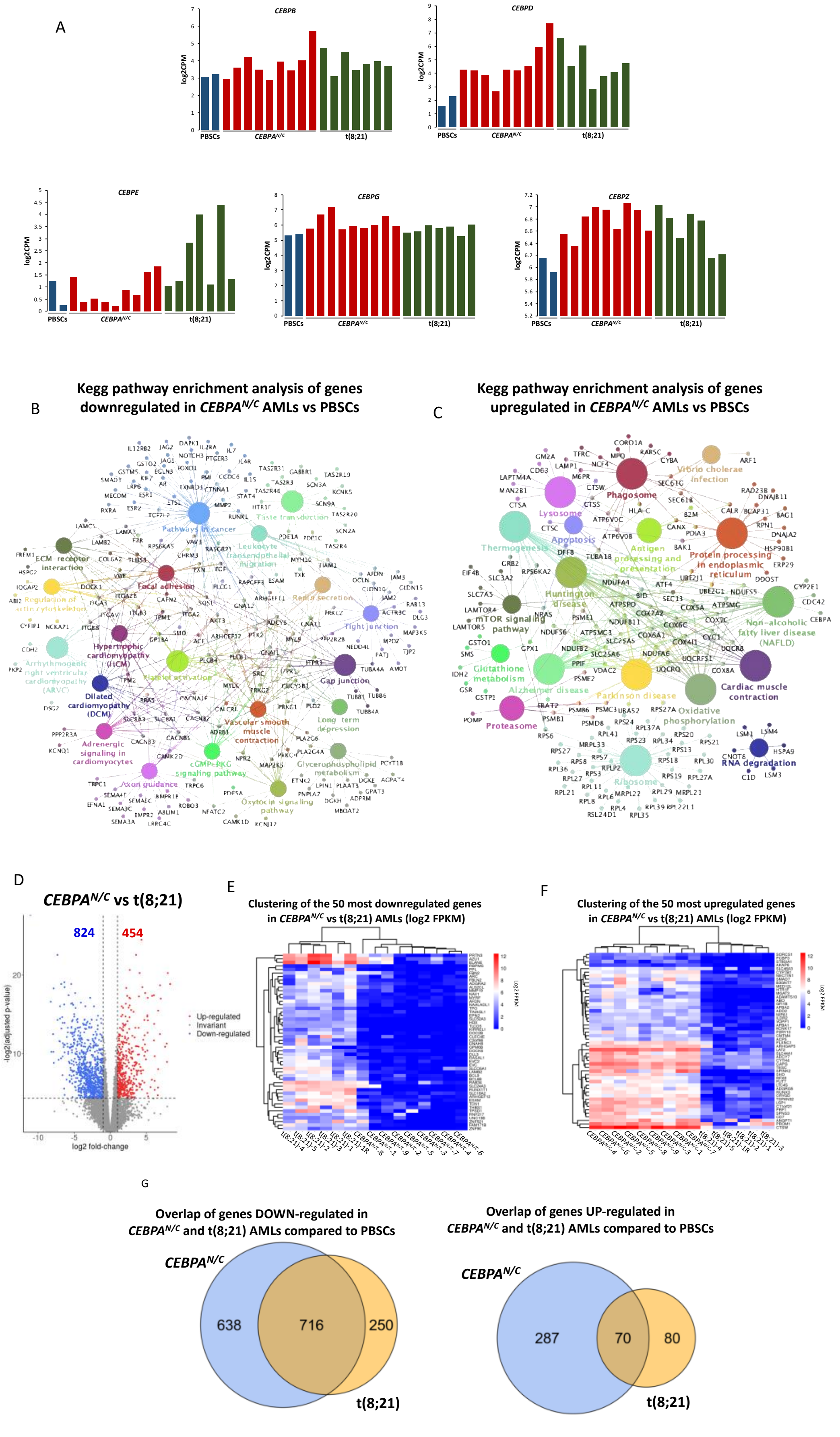
A
CEBPB
CEBPD
CEBPE
CEBPG
CEBPZ
log2CPM
PBSCs
CEBPA^N/C
t(8;21)
B
Kegg pathway enrichment analysis of genes downregulated in CEBPA^N/C AMLs vs PBSCs
Pathways in cancer
Taste transduction
Leukocyte transendothelial migration
ECM-receptor interaction
Focal adhesion
Regulation of actin cytoskeleton
Renin secretion
Tight junction
Hypertrophic cardiomyopathy (HCM)
Arrhythmogenic right ventricular cardiomyopathy (ARVC)
Platelet activation
Gap junction
Dilated cardiomyopathy (DCM)
Vascular smooth muscle contraction
Long-term depression
Adrenergic signaling in cardiomyocytes
cGMP-PKG signaling pathway
Glycerophospholipid metabolism
Axon guidance
Oxytocin signaling pathway
C
Kegg pathway enrichment analysis of genes upregulated in CEBPA^N/C AMLs vs PBSCs
Vibrio cholerae infection
Phagosome
Lysosome
Apoptosis
Thermogenesis
Antigen processing and presentation
Protein processing in endoplasmic reticulum
Huntington disease
mTOR signaling pathway
Non-alcoholic fatty liver disease (NAFLD)
Glutathione metabolism
Alzheimer disease
Parkinson disease
Cardiac muscle contraction
Oxidative phosphorylation
Proteasome
RNA degradation
Ribosome
D
CEBPA^N/C vs t(8;21)
824
454
-log2(adjusted p-value)
log2 fold-change
Up-regulated
Invariant
Down-regulated
E
Clustering of the 50 most downregulated genes in CEBPA^N/C vs t(8;21) AMLs (log2 FPKM)
F
Clustering of the 50 most upregulated genes in CEBPA^N/C vs t(8;21) AMLs (log2 FPKM)
Log2 FPKM
G
Overlap of genes DOWN-regulated in CEBPA^N/C and t(8;21) AMLs compared to PBSCs
CEBPA^N/C
638
716
250
t(8;21)
Overlap of genes UP-regulated in CEBPA^N/C and t(8;21) AMLs compared to PBSCs
CEBPA^N/C
287
70
80
t(8;21)

Figure S2: Differential gene expression analysis comparing *CEBPA*$^{N/C}$ and t(8;21) blasts with each other and with healthy PBSCs (A) mRNA expression levels of *CEBP* genes in PBSCs and *CEBPA*$^{N/C}$ and t(8;21) blasts. (B) and (C) KEGG pathway enrichment analysis of genes downregulated (B) and upregulated (C) in *CEBPA*$^{N/C}$ AMLs compared to PBSCs. (D) Volcano plot of genes deregulated in *CEBPA*$^{N/C}$ compared to t(8;21). Each dot represents a gene, the log2 fold-change indicates the mean expression level for each gene across all samples. Blue dots represent significantly downregulated genes (log2 fold-change < -1, Benjamini-Hochberg adjusted p-value < 0.05); red dots represent significantly upregulated genes (log2 fold-change > 1, Benjamini- Hochberg adjusted p-value < 0.05). (E) and (F) Hierarchical clustering of expression values for the 50 most downregulated (E) and upregulated (F) genes in *CEBPA*$^{N/C}$ compared to t(8;21). (G) and (H) Venn diagrams showing the overlap of genes commonly downregulated (G) and upregulated (H) in *CEBPA*$^{N/C}$ and t(8;21) AMLs compared to PBSCs.

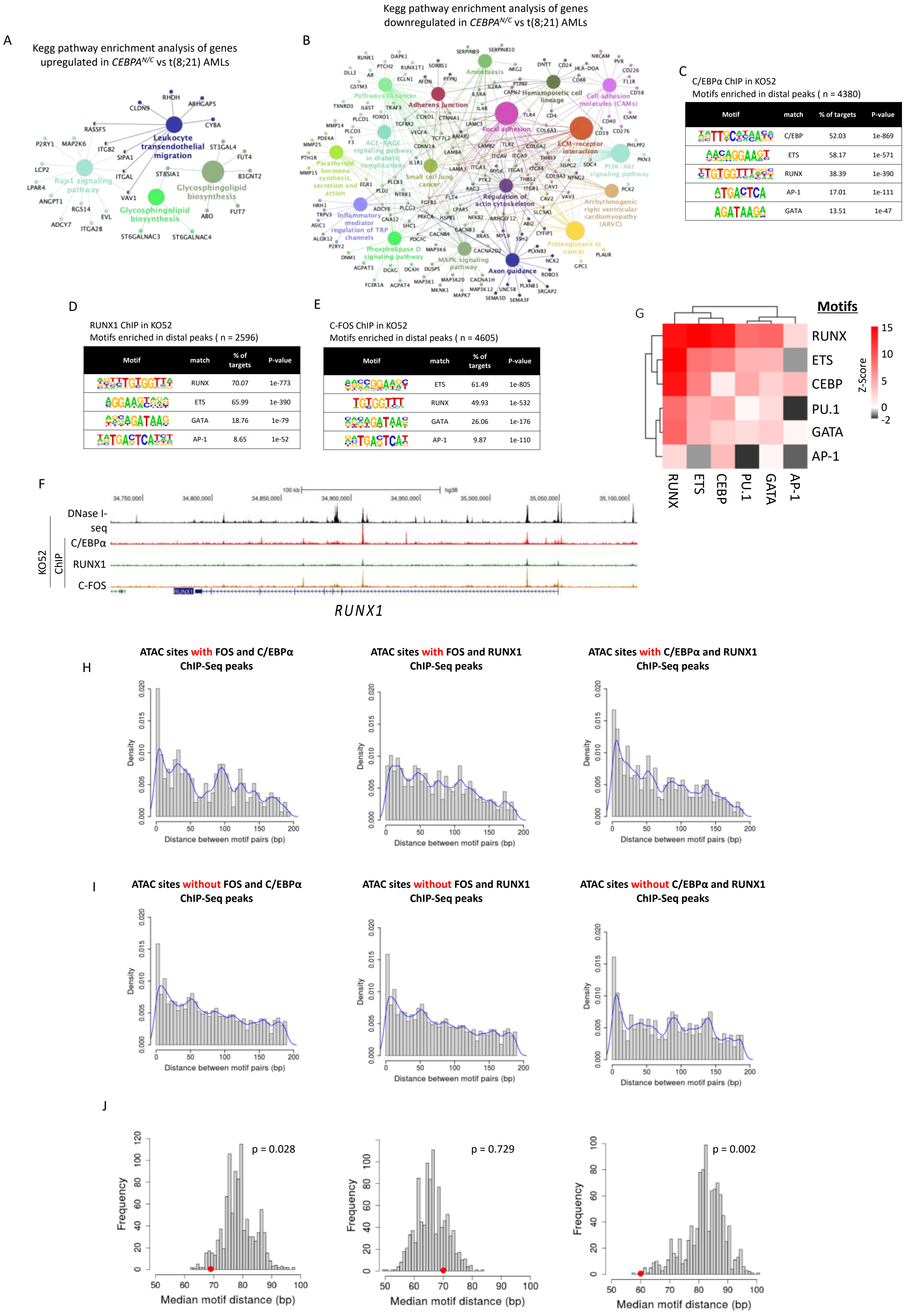
A
Kegg pathway enrichment analysis of genes upregulated in CEBPA^N/C vs t(8;21) AMLs
Leukocyte transendothelial migration
Rap1 signaling pathway
Glycosphingolipid biosynthesis
B
Kegg pathway enrichment analysis of genes downregulated in CEBPA^N/C vs t(8;21) AMLs
C
C/EBPα ChIP in KO52
Motifs enriched in distal peaks (n = 4380)
Motif | match | % of targets | P-value
C/EBP | 52.03 | 1e-869
ETS | 58.17 | 1e-571
RUNX | 38.39 | 1e-390
AP-1 | 17.01 | 1e-111
GATA | 13.51 | 1e-47
D
RUNX1 ChIP in KO52
Motifs enriched in distal peaks (n = 2596)
Motif | match | % of targets | P-value
RUNX | 70.07 | 1e-773
ETS | 65.99 | 1e-390
GATA | 18.76 | 1e-79
AP-1 | 8.65 | 1e-52
E
C-FOS ChIP in KO52
Motifs enriched in distal peaks (n = 4605)
Motif | match | % of targets | P-value
ETS | 61.49 | 1e-805
RUNX | 49.93 | 1e-532
GATA | 26.06 | 1e-176
AP-1 | 9.87 | 1e-110
G
Motifs
RUNX
ETS
CEBP
PU.1
GATA
AP-1
Z-Score
F
DNase I-seq
KO52
ChIP
C/EBPα
RUNX1
C-FOS
RUNX1
H
ATAC sites with FOS and C/EBPα ChIP-Seq peaks
ATAC sites with FOS and RUNX1 ChIP-Seq peaks
ATAC sites with C/EBPα and RUNX1 ChIP-Seq peaks
Density
Distance between motif pairs (bp)
I
ATAC sites without FOS and C/EBPα ChIP-Seq peaks
ATAC sites without FOS and RUNX1 ChIP-Seq peaks
ATAC sites without C/EBPα and RUNX1 ChIP-Seq peaks
Density
Distance between motif pairs (bp)
J
p = 0.028
p = 0.729
p = 0.002
Frequency
Median motif distance (bp)

Figure S3: **Motif spacing analysis demonstrating colocalization of AP-1, C/EBP and RUNX motifs.** (A) and (B) KEGG pathway enrichment analysis of genes up (A) and down (B) regulated in *CEBPA*$^{N/C}$ compared to t(8;21) AMLs. Related to Fig.3. (C-E) Motifs enrichment in C/EBPα (C), RUNX1 (D) and c-FOS (E) distal ChIP peaks in KO52 cell line. (F) UCSC genome browser screenshot depicting C/EBPα (red), RUNX1 (green) and c-FOS (ochre) binding patterns around the *RUNX1* gene locus in KO52. (G) Co-localization for selected motifs for sites bound by C/EBPα, RUNX1 and c-FOS in KO52 cells. (H) and (I) Distribution of the distances between C/EBP and AP-1, RUNX1 and AP-1 and C/EBP and RUNX motifs in open chromatin regions bound (H) or not bound (I) by the indicated transcription factors as determined by ChIP-seq. (J) Median distance between motif pairs in 1000 random peak sets compared to peaks with ChIP-seq signal.

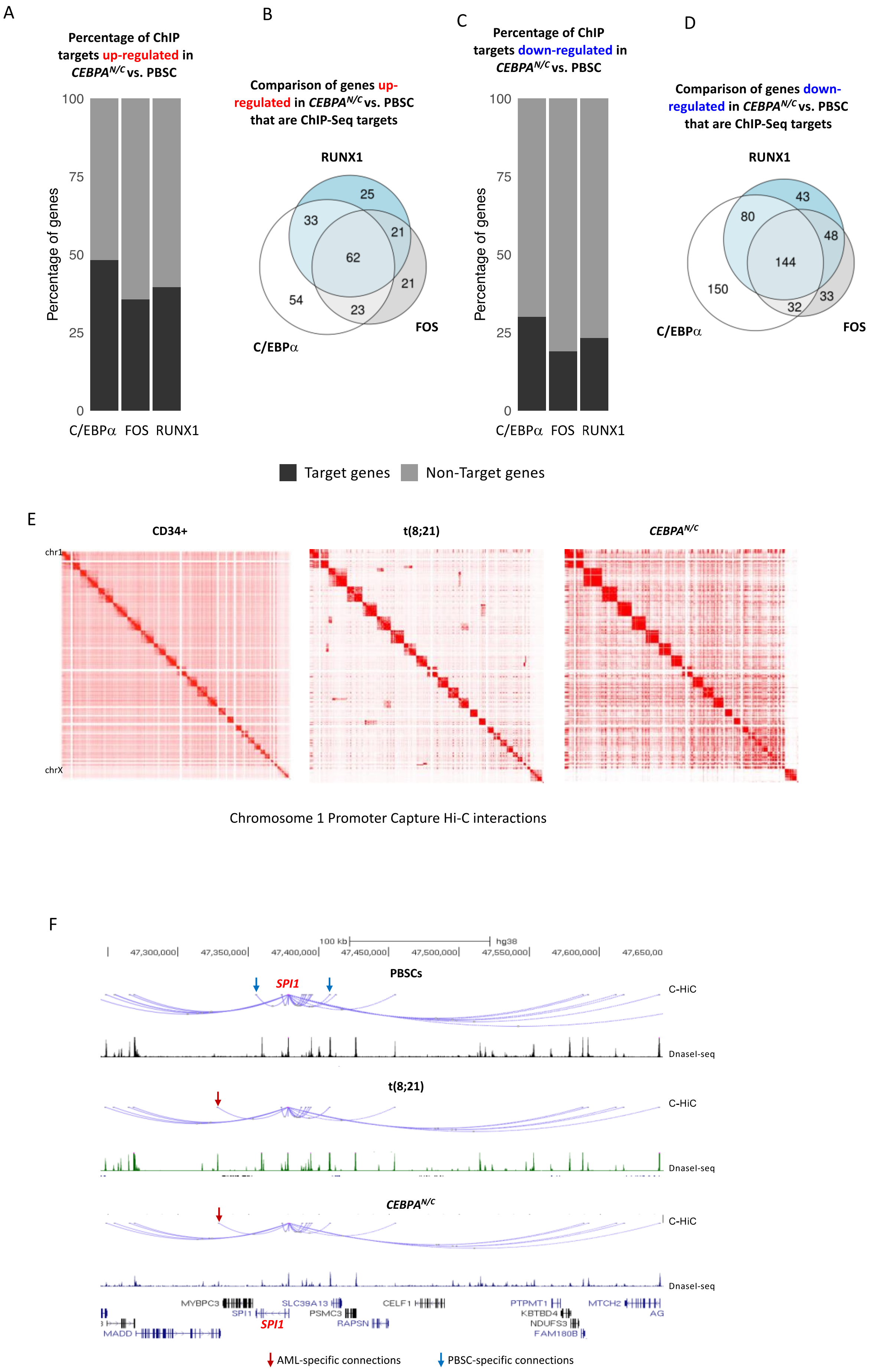
A
Percentage of ChIP targets up-regulated in CEBPA^N/C vs. PBSC
100
75
50
25
0
Percentage of genes
C/EBPα FOS RUNX1
B
Comparison of genes up-regulated in CEBPA^N/C vs. PBSC that are ChIP-Seq targets
RUNX1
25
33
21
62
21
54
23
FOS
C/EBPα
C
Percentage of ChIP targets down-regulated in CEBPA^N/C vs. PBSC
100
75
50
25
0
Percentage of genes
C/EBPα FOS RUNX1
D
Comparison of genes down-regulated in CEBPA^N/C vs. PBSC that are ChIP-Seq targets
RUNX1
43
80
48
144
150
33
32
C/EBPα
FOS
Target genes
Non-Target genes
E
CD34+
t(8;21)
CEBPA^N/C
chr1
chrX
Chromosome 1 Promoter Capture Hi-C interactions
F
100 kb
hg38
47,300,000
47,350,000
47,400,000
47,450,000
47,500,000
47,550,000
47,600,000
47,650,00
PBSCs
SPI1
C-HiC
DnaseI-seq
t(8;21)
C-HiC
DnaseI-seq
CEBPA^N/C
C-HiC
DnaseI-seq
MYBPC3
SLC39A13
CELF1
PTPMT1
MTCH2
SPI1
PSMC3
KBTBD4
AG
SPI1
RAPSN
NDUFS3
MADD
FAM180B
AML-specific connections
PBSC-specific connections

Figure S4: *CEBPA$^{N/C}$* AML sub-type specifically expressed genes contain a large percentage of C/EBPα, RUNX1 and c-FOS ChIP targets and are associated with myeloid differentiation. (A) Percentage of ChIP targets upregulated in *CEBPA$^{N/C}$* AMLs compared to PBSCs. (B) Overlap of C/EBPα, RUNX1 and c-FOS ChIP targets upregulated in *CEBPA$^{N/C}$* AMLs compared to PBSCs. (C) Percentage of ChIP targets downregulated in *CEBPA$^{N/C}$* AMLs compared to PBSCs. (D) Overlap of C/EBPα, RUNX1 and c-FOS ChIP targets downregulated in *CEBPA$^{N/C}$* AMLs compared to PBSCs. (E) Contact matrix across all chromosomes for healthy blasts (left), t(8;21) (middle) and *CEBPA$^{N/C}$* (right) leukemic blasts at 10Mb resolution. Colour intensity represents interaction frequency. (F) UCSC genome browser screenshot depicting *SP1* promoter interactions with close cis-regulatory elements in healthy (top), *CEBPA$^{N/C}$* (middle) and t(8;21) (bottom) blasts. A blue arrow indicates a PBSC-specific interaction, a red arrow indicates an AML-specific interaction.

A

Dataset	# interactions
CD34+	111883
CEBPA$^{N/C}$	117243
t(8;21)	129971

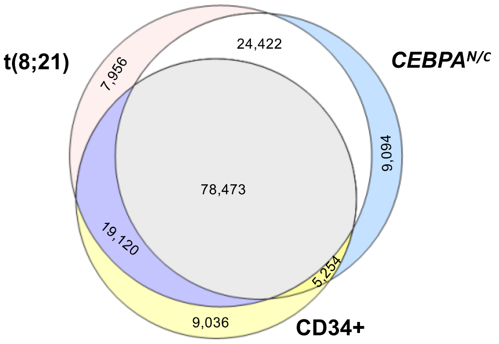

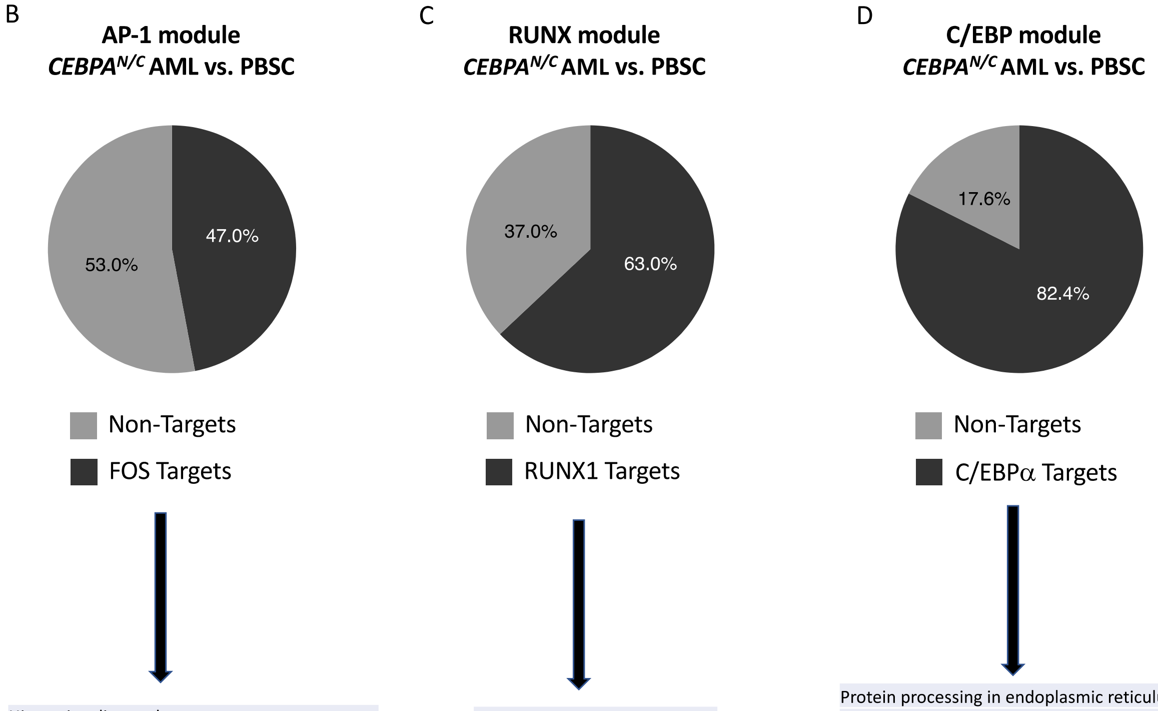

Hippo signaling pathway
Insulin resistance
Insulin signaling pathway
cGMP-PKG signaling pathway
Ubiquitin mediated proteolysis
Phagosome
Wnt signaling pathway
Platelet activation
Apelin signaling pathway
T cell receptor signaling pathway
Th1 and Th2 cell differentiation
Signaling pathways regulating pluripotency of stem cells
Apoptosis
Osteoclast differentiation
Leukocyte transendothelial migration
HIF-1 signaling pathway
Toll-like receptor signaling pathway

Chemokine signaling pathway
Tight junction
Phagosome

Protein processing in endoplasmic reticulum
NOD-like receptor signaling pathway
Leukocyte transendothelial migration
HIF-1 signaling pathway
Fanconi anemia pathway
Insulin resistance

Module associated KEGG pathways

Figure S5: **GRN derived AP-1, RUNX1 and C/EBP module genes are bound by their respective transcription factor.** (A) Table reporting the number of interactions identified for each cell type analysed. Right panel: Venn diagram showing the number of interactions shared among

the analysed samples. (B-D top) Pie charts showing the percentage of target genes identified in the AP-1 (B), RUNX (C) and C/EBP (D) network modules validated by ChIP-seq. At the bottom, KEGG pathway enrichment analysis of target genes identified in the AP-1, RUNX and C/EBP network modules. More details can be bound in Supplementary dataset 3.

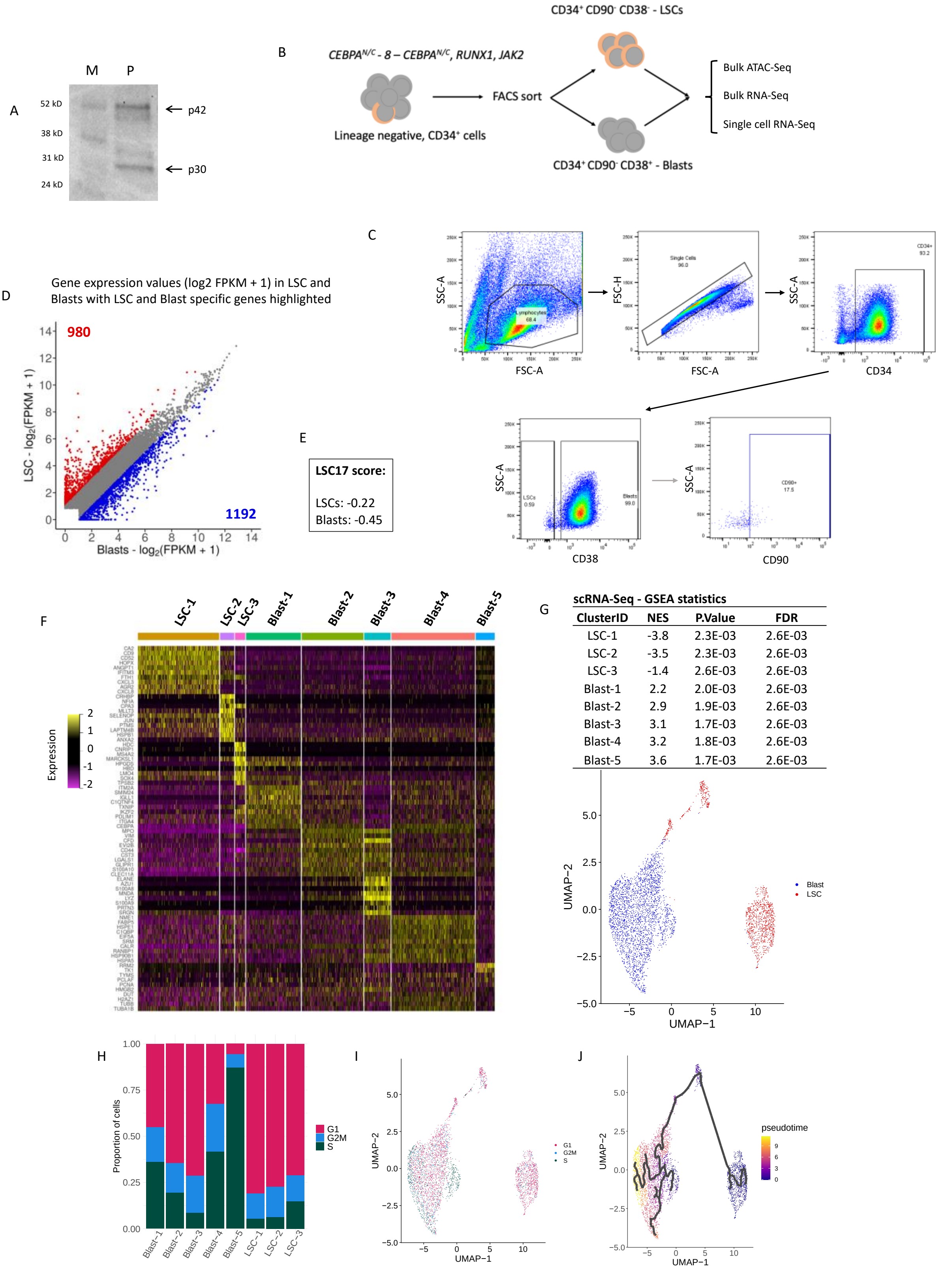

Figure S6: Single cell RNA-Seq analysis of *CEBPA*$^{N/C}$ leukemic stem and blast cells. (A) C/EBPα p42 and p30 isoform expression assessed by western blot in patient *CEBPA*$^{N/C}$ -8. M: Size marker, P: sample. (B) Experimental overview. (C) Sorting strategy for LSCs and Blast cells.

Viable cells were gated on FSC-A vs SSC-A, followed by doublet discrimination gating on FSC-A vs FSC-H. CD34 positive cells were identified, and subsequently gated for CD38+ (blasts) and CD38- (LSCs). CD90 was assessed in both blasts and LSCs - but not used to sort by as levels were similar in both populations. (D) Log_2 transformed FPKM gene expression values in LSCs and blasts. Each dot represents a gene, LSC-specific genes are highlighted in red, blast-specific in blue. (E) 17-gene LSC score (LSC17) of the purified LSC and blast populations determined as in (1). (F) Heatmap depicting the top 10 most upregulated marker genes identified in each scRNA-seq cluster. (G) Gene Set Enrichment Analysis statistics of cluster marker genes identified in scRNA-seq populations with the resulting UMAP clusters plotted underneath. (H) Histogram showing the proportion of cells in each cell cycle phase within each cluster as identified by the expression of cell-cycle regulated genes. (I) Expression of cell-cycle phase-specific genes (G0-G1, G2-M, S) projected on the UMAP map of scRNA populations. (J) Monocle pseudo-time trajectory of LSC and blast cells projected on the UMAP map of scRNA clusters. Cells are coloured according to their pseudo-time value.

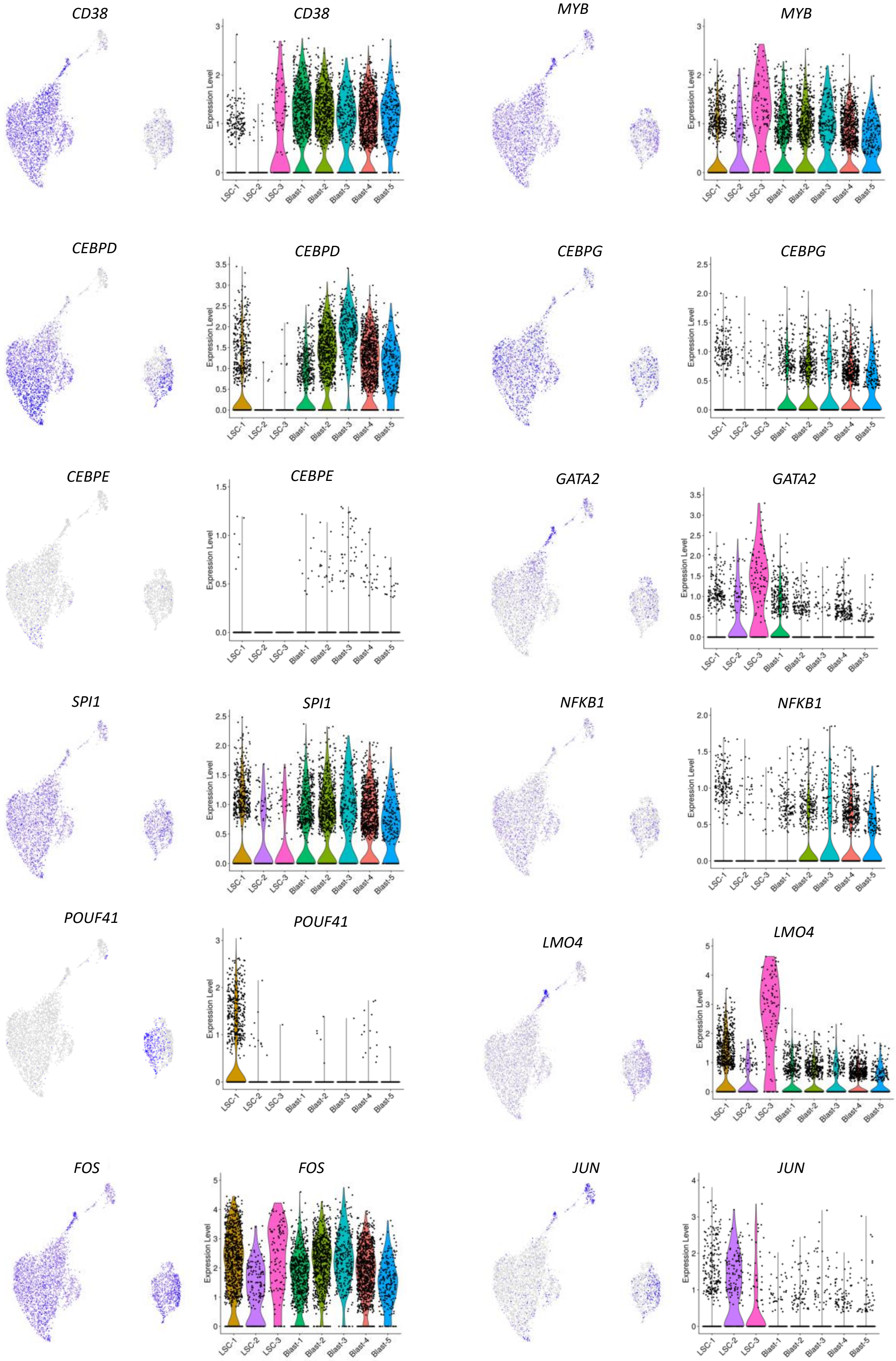
CD38
CD38
MYB
MYB
CEBPD
CEBPD
CEBPG
CEBPG
CEBPE
CEBPE
GATA2
GATA2
SPI1
SPI1
NFKB1
NFKB1
POUF41
POUF41
LMO4
LMO4
FOS
FOS
JUN
JUN
Expression Level
LSC-1
LSC-2
LSC-3
Blast-1
Blast-2
Blast-3
Blast-4
Blast-5

Figure S7: Expression of marker and regulator genes in *CEBPA*$^{N/C}$ LSC and blast cells. Expression of indicated genes projected on the UMAP map. Colour intensity represents expression data log2 normalized unique molecular identifier (UMI) counts.

Figure S8

A

LSC specific marker genes from figure 7

GeneID	prop.LSC.exprs	prop.Blast.exprs	avg.logFC	P.Value	Adj.P.Value
LMO4	0.54	0.39	1.19	7.2E-61	1.0E-56
SOX4	0.84	0.81	0.94	4.6E-66	6.3E-62
POU4F1	0.29	0.01	0.76	9.7E-175	1.3E-170
JUN	0.29	0.10	0.76	3.3E-62	4.6E-58

Blast specific marker genes from figure 7

GeneID	prop.Blast.exprs	prop.LSC.exprs	avg.logFC	P.Value	Adj.P.Value
CEBPA	0.95	0.38	1.30	0	0
CEBPD	0.78	0.28	0.59	1.1E-140	1.5E-136
NFKB1	0.32	0.17	0.05	5.3E-13	7.3E-09

B

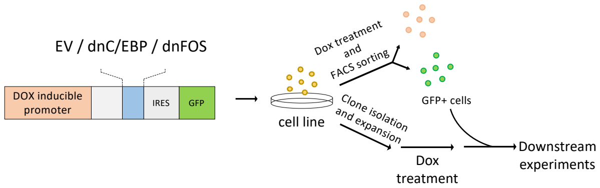

C

Gained sites – n = 702

Motif	Match	%sites	p-value
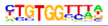	RUNX	47.72	1e-49
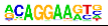	ETS	47.29	1e-61
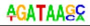	GATA	38.03	1e-65
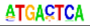	AP-1	30.48	1e-58

Lost sites – n = 420

Motif	Match	%sites	p-value
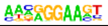	ETS	40.71	1e-71
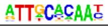	CEBP	40.00	1e-96
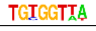	RUNX	33.81	1e-29

D

Genes differentially expressed in KO52-dnC/EBP vs KO52-EV

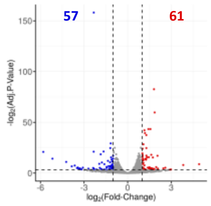

dnC/EBP – Up-Regulated genes

ADAMTS14	GATA1	SERPINB9
ADGRG1	H3C14	SPTA1
ADRB1	HMCN1	SPTBN2
APOL3	IL21R	STON2
APOL4	ITGB5	SYTL4
BHLHE40	JUN	TAFA2
BRSK1	KRBA1	TNF
C20orf203	LIF	TNFRSF19
CASTOR2	MAP1A	TNFRSF4
CAVIN1	MS4A6A	TPSB2
CCR2	MUC4	TPSD1
CCR5	NCR3	TRAF1
CD36	NFATC4	TSPAN15
CFH	NTRK1	TUBA4A
CISH	OSM	UBASH3A
COL1A2	PIEZO2	VWF
COLQ	PIM1	ZFPM1
CSMD3	PTGIR	ZNF185
FKBP10	PTGS2	ZNF469
FOSB	RIPOR3	
FSD1	RSAD2	

dnC/EBP – Down-Regulated genes

ADGRE1	FGFR1	NRG4
ALG14	GLB1L2	PKIA
ARHGAP42	GPR141	PRTN3
ARSG	GPR35	RAG1
BTBD11	HGF	RNASE2
C1orf162	INHBA	RNASE3
C5orf34	KBTBD11	RRH
CA2	LCT	SAMD13
CCDC85C	LGR4	SLC25A21
CD38	LIN28B	ST18
CLC	LINC00672	ST3GAL6
CRYBG1	LPAR4	TMEM200A
CTSG	LRP6	TMEM243
CXCR4	MAMDC2	TNFAIP8L3
CXXC4	MDP1	USP44
CYP2U1	MMP2	
DBNDD1	MPO	
DLGAP3	MT-ATP8	
ENPP2	MYO1B	
FAM72D	NLRC4	
FCGR1A	NPHP1	

E

dnC/EBP – Up-Regulated genes

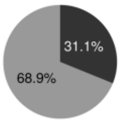

dnC/EBP – Down-Regulated genes

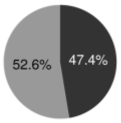

Non-target genes

C/EBPα target genes

Figure S8: Perturbation of the *CEBPA*$^{N/C}$ GRN. (A) Analysis of the statistical significance of the distribution of expression of the indicated marker genes between LCSs and Blast populations. (B) Experimental overview. (C) Motifs enriched in hypersensitive sites deregulated in KO52 cells expressing dnC/EBP.(D) Volcano plot of genes deregulated in KO52 cells expressing dnC/EBP compared to the empty vector control. Blue dots represent significantly downregulated genes (log2 fold-change < -1, Benjamini-Hochberg adjusted p-value < 0.05); red dots represent significantly upregulated genes (log2 fold-change > 1, Benjamini-Hochberg adjusted p-value < 0.05). Right panel: List of up and down-regulated genes. (E) Pie charts showing the percentage of genes in (D) that are C/EBPα targets. (E) Motif enriched in dnFOS-specific and control-specific hypersensitive sites.

Figure S9

A

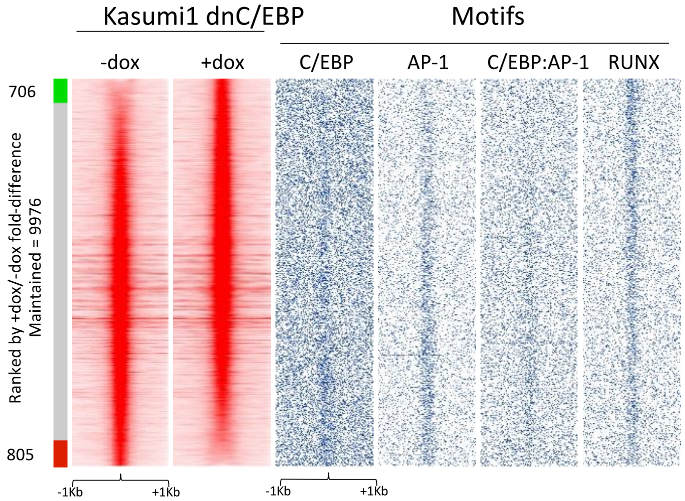

B

Motifs enriched in DHSs gained upon dnC/EBP expression

Logo	% of targets	p-value	Match
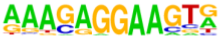	24.32%	1e-65	PU.1
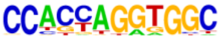	8.71%	1e-33	CTCF
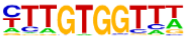	20.15%	1e-32	RUNX

C

Motifs enriched in DHSs lost upon dnC/EBP expression

Logo	% of targets	p-value	Match
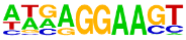	26.86%	1e-46	PU.1
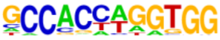	7.28%	1e-28	CTCF
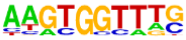	13.92%	1e-18	RUNX
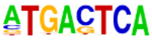	12.46%	1e-14	AP-1
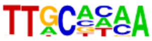	21.04%	1e-13	C/EBP

D

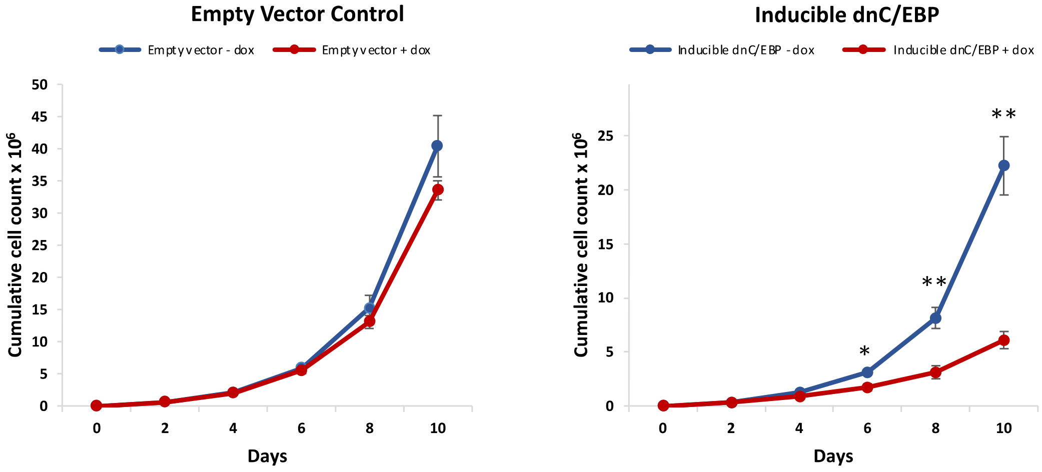

Kasumi-1 cells (t(8;21))

Figure S9: C/EBP family members are important for t(8;21) growth. (A) Density plots showing the DNaseI-seq profile of Kasumi-1 cells expressing dnC/EBP or a control vector across a 2kb window. Data are ranked by normalized tag counts of dnC/EBP expressing cells over control. TF binding motifs projected against hypersensitive sites are plotted alongside. (B) and (C) Motif enriched in dnC/EBP-specific (B) and control-specific (C) hypersensitive sites. (D) Cumulative cell growth time course of Kasumi-1 cells expressing an inducible control vector or dnC/EBP with or without doxycycline (N = 3). Error bars represent the sample standard deviation. P values were calculated using a two-tailed t-test.

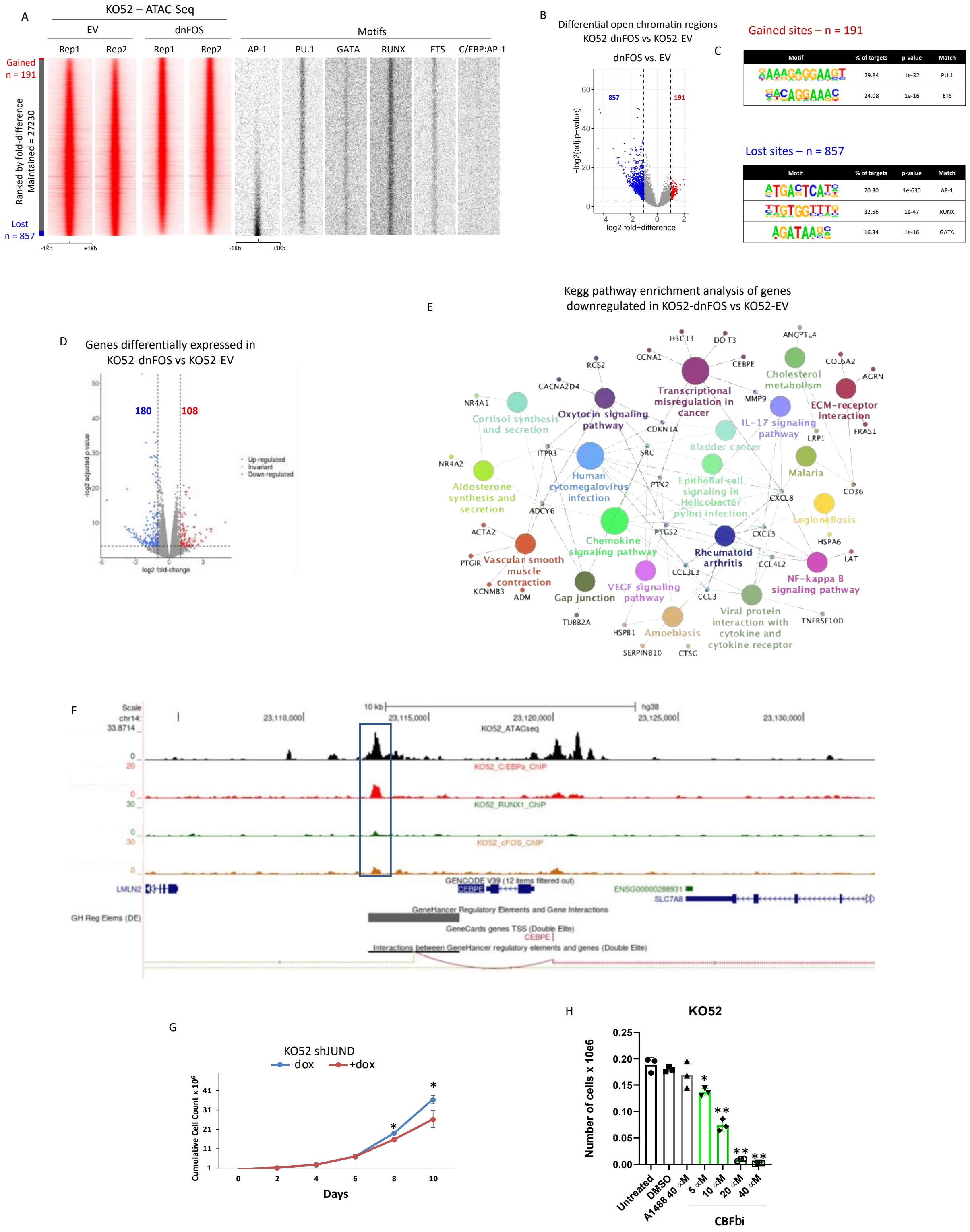

Figure S10: AP-1 and RUNX1 regulate *CEBPA*$^{N/C}$ growth. (A) Density plots showing the ATAC-seq profile of KO52 cells expressing dnFOS or a control vector across a 2kb window (2 replicates). Data are ranked by normalized tag counts of dnFOS expressing cells over control. TF binding motifs projected against hypersensitive sites are plotted alongside. (B) Volcano

plot of hypersensitive sites deregulated in KO52 cells expressing dnFOS compared to control. Blue and red dots represent significantly downregulated (log2 fold-change < -1, Benjamini-Hochberg adjusted p- value < 0.05) and significantly upregulated (log2 fold-change > 1, Benjamini-Hochberg adjusted p-value < 0.05) hypersensitive sites. (C) Motif enriched in dnFOS-specific and control-specific hypersensitive sites. (D) Volcano plot of genes deregulated in KO52 cells expressing dnFOS compared to control. Each dot represents a gene, the log2 fold-change indicates the mean expression level for each gene across all samples. Blue dots represent significantly downregulated genes (log2 fold-change < -1, Benjamini-Hochberg adjusted p-value < 0.05); red dots represent significantly upregulated genes (log2 fold-change > 1, Benjamini- Hochberg adjusted p-value < 0.05). (E) KEGG pathway enrichment analysis of genes downregulated in KO52 cells expressing dnFOS compared to control. (F) UCSC genome browser screenshot depicting the *CEBPE* gene locus with nearby cis-regulatory elements and the genome track of hypersensitive site (black) and C/EBPα (red), RUNX1 (green) and c-FOS (ochre) ChIP peaks. A *CEBPE* enhancer bound by all three TFs is highlighted. (G) Cumulative growth time course of KO52 cells expressing an inducible shRNA targeting *JUND* with or without doxycycline (N=3). (H) Cumulative growth of KO52 cells after one week of CBFβi treatment at the indicated concentrations of CBFβ inhibitor (N=3).

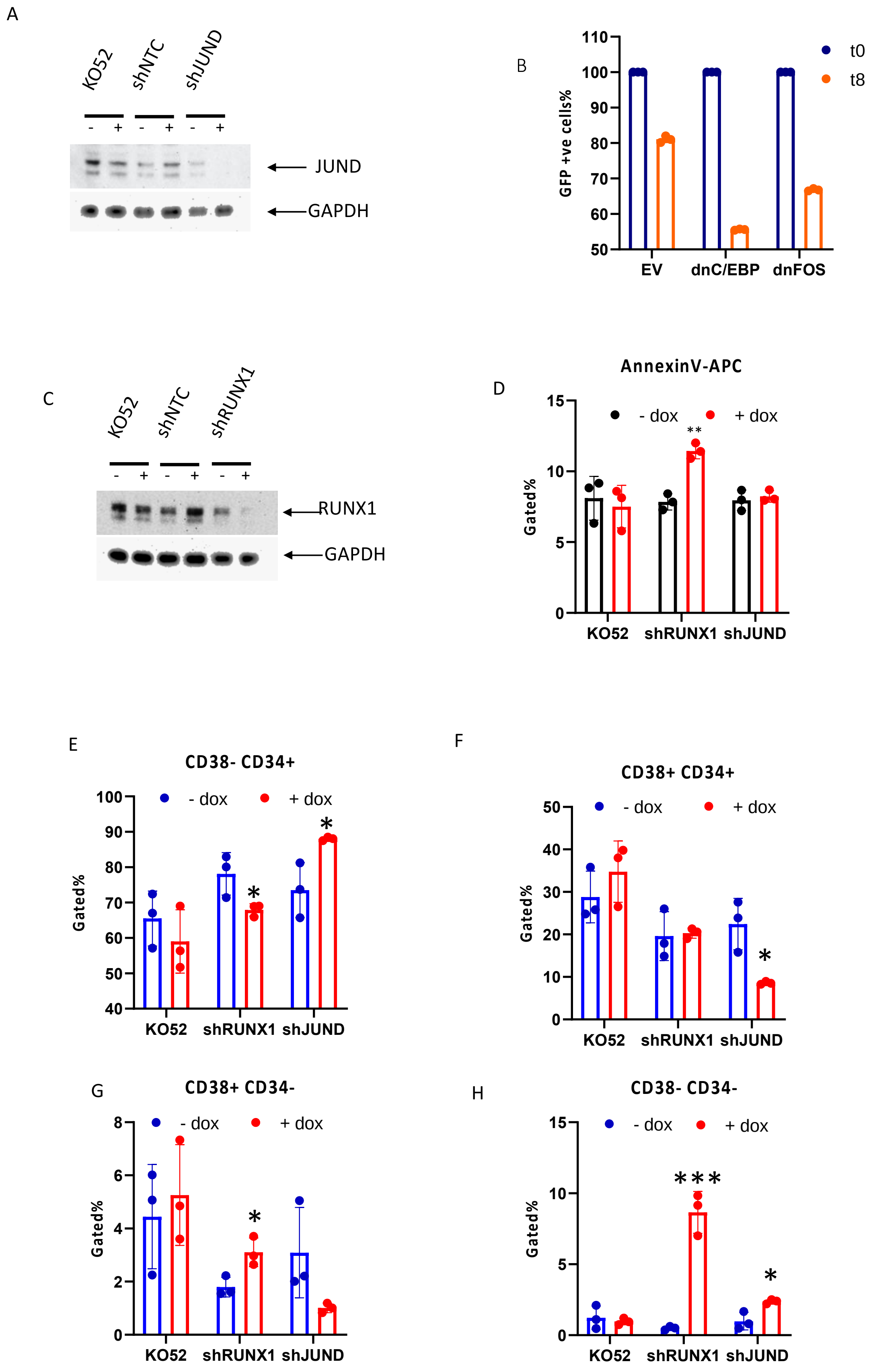
A
KO52
shNTC
shJUND
- + - + - +
JUND
GAPDH
B
GFP +ve cells%
110
100
90
80
70
60
50
EV
dnC/EBP
dnFOS
t0
t8
C
KO52
shNTC
shRUNX1
- + - + - +
RUNX1
GAPDH
D
AnnexinV-APC
15
10
5
0
Gated%
- dox
+ dox
**
KO52
shRUNX1
shJUND
E
CD38- CD34+
100
90
80
70
60
50
40
Gated%
- dox
+ dox
*
*
KO52
shRUNX1
shJUND
F
CD38+ CD34+
50
40
30
20
10
0
Gated%
- dox
+ dox
*
KO52
shRUNX1
shJUND
G
CD38+ CD34-
8
6
4
2
0
Gated%
- dox
+ dox
*
KO52
shRUNX1
shJUND
H
CD38- CD34-
15
10
5
0
Gated%
- dox
+ dox
***
*
KO52
shRUNX1
shJUND

Figure S11: AP-1 and RUNX1 regulate *CEBPA*$^{N/C}$ cellular states

(A) and (C) JUND (A) and RUNX1 (C) proteins expression in KO52 cells wt or expressing an inducible shRNA targeting *JUND* (B) or *RUNX1* (C) with and without doxycycline. (B) Percentage of GFP+ cells after 8 days of EV, dnC/EBP or dnFOS induction. (D) Percentage of Annexin V positive KO52 wt or expressing an inducible shRNA targeting *JUND* or *RUNX1* with or without doxycycline after 10 days (N=3). Error bars represent the sample standard deviation. (E-F) FACS analysis assessing CD38 and CD34 expression in cells in (D). Error bars represent the sample standard deviation. P values were calculated using a two-tailed t-test.

Supplementary Methods

Isolation of hematopoietic stem/progenitor cells (HSPCs)

Patient CD34+ or CD117+ hematopoietic progenitors were isolated with CD34 MicroBead Kit, human or CD117 MicroBead Kit, human (Miltenyi-Biotech) following manufacturer's instructions. To increase cell purity, the eluted fraction was applied onto a new LS column and the whole procedure repeated.

Inducible plasmid generation

The doxycycline inducible pCW57.1-dnC/EBP plasmid was generated using Gateway Gene Cloning (Thermo Fisher Scientific), according to manufacturer's instructions. The CMV500 A-C/EBP plasmid containing the dnC/EBP insert was a gift from Charles Vinson, National Cancer Institute, Bethesda, USA (2). dnC/EBP cDNA was PCR amplified (with Phusion DNA polymerase (New England Biolabs) using primers containing SalII and NotI restriction enzymes overhangs and cloned into pENTR-IRES-GFP (a modification of the Invitrogen pENTR plasmid constructed by Benjamin Edginton-White, University of Birmingham, UK) digested with 30 units of SalI-HF (New England Biolabs), 30 units of NotI-HF (New England Biolabs). The dnC/EBP insert was then transferred into the Tet-on pCW57.1 backbone (Addgene plasmid #41393) by using the Gateway Gene Cloning System. The pCW57.1 vector containing the dnFOS insert was generated by Dr Sandeep Potluri (University of Birmingham) following the same protocol (3). The doxycycline inducible pLenti.TTMPVIV-(N) expressing shRNAs targeting *RUNX1* or *JUND* were generated by Dr Sophie Kellaway as previously described in (4).

shRNA Target	Guide sequence
RUNX1	TCCACTGTGATTTTGATGGCTC
JUND	TTCACTTTCTCTTCCAGGCGC

Lentiviral production and cell infection

Lentiviral particles containing pCW57.1-dnC/EBP / dnFOS or the empty vector were generated in HEK293T cells using calcium-phosphate transfection. Cells were cultured in DMEM

supplemented with 10% foetal calf serum (FCS), 5% Penicillin-Streptomycin (Pen-Strep), 5% L-Glutamine (L-Glu) and 5% Sodium Pyruvate and seeded to achieve about 30-40% confluency at the time of transfection. 12 µg of pCW57.1-dnC/EBP or empty vector were transfected with 0.6 ug of REV, 0.6 ug of GAG/POL, 1.2 ug of VSV-G and 0.6 ug of TAT packaging plasmids. Viral supernatant was harvested at 24, 36, 48, 60 and 72h and concentrated with Centricon Plus-70 columns (Merk-Millipore) using manufacturer's instructions. Concentrated viral particles were filtered through 0.45 um filter and stored at -80°C or immediately used to infect recipient cells. To carry out the spin infection, 1 x 10^6 Kasumi-1 or KO52 cells were plated in 6-well plates with the viral supernatant and 8 ug/mL of polybrene, and centrifuged for 1h 30m at 1500 x g at 32°C. After infection cells were incubated at 37 °C for 12 hours and then resuspended in fresh media. Few days after infection, cells were cultured in medium supplemented with puromycin 1.5 ug/mL and 2ug/mL for 5 days, allowing the selection of cells positive for pCW57.1-dnC/EBP. Single clones were then isolated from the bulk Kasumi-1 population, expanded and tested for leakiness and induction of dnC/EBP measuring both GFP and FLAG expression through both FACS and RT-qPCR. Despite several attempts, it was not possible to expand KO52 single cell clones with either vector and the bulk population was used for downstream experiments. Dominant negative peptides expression was induced by adding 2 µg/mL of doxycycline to culture medium.

Proliferation assay

5x10^5 cells/mL for each condition were seeded and treated with 2 µg/mL of doxycycline where appropriate. Cell viability was assessed by using Trypan Blue (Sigma-Aldrich) and 5x10^5 cells/mL were re-seeded in media with or without doxycycline every two days up to ten days. KO52 cells expressing the inducible dnC/EBP and dnFOS were induced prior to the start of the time-course and GFP+ FACS sorted cells were used to set up the assay as described above.

Pharmacological inhibition of RUNX in primary blasts

3x10^5 cells/mL were seeded and treated with 5, 10, 20 and 40 µM of the CBFβ inhibitor (5). After 7 days, cell viability was assessed by using Trypan Blue (Sigma-Aldrich).

RNA extraction and Real-Time RT-qPCR

Total RNA was extracted with NucleoSpin RNA (Machery-Nagel) and 1 ug of nucleic acid was retrotranscribed using SuperScriptTM II Reverse Transcriptase kit (Thermo Fisher) and 0.5 µg of Oligo(dT) primers (Promega), according to the manufacturer's instruction. Target gene expression was calculated using target specific standard curve obtaining a sample cDNA in water at the serial dilution 1:10, 1:50, 1:250, 1:1250 and 1:6250. Sample cDNA was diluted 1:100 in water prior to quantification. Real time PCRs were performed using a 2x dilution of SYBR® Green PCR Mix (Applied Biosystems) and 100 nM forward and reverse primers. 2.5 uL of standard or sample diluted as specified above were added in a final volume of 10 uL. *GAPDH* was used as reference gene for relative gene expression calculation Analysis were performed on an ABI 7500 real-time PCR system using StepOneTM Plus software.

ATAC-seq and Data analysis

Fragment transposition

ATAC libraries were generated following the Omni-ATAC protocol from (6). $50x10^3$ viable cells were pelleted in a fixed angle centrifuge at 500xg at 4°C for 5 min, resuspended in 50 µL of cold ATAC-Resuspension Buffer (RSB) (1M Tris-HCl pH 7.4, 5M NaCl, 1M $MgCl_2$ in sterile H_2O) containing 0.1% NP40 (Sigma-Roche), 0.1% Tween-20 (Sigma-Roche), 0.01% Digitonin (Promega) and incubated in ice for 3 min. Lysis was washed out by adding 1 mL of cold ATAC-RSB containing 0.1% Tween-20 (Sigma/Roche) only. Nuclei were pelleted at 500xg at 4°C for 10 min and resuspended in 50 µL of transposition mix (25 µL of 2x TD buffer (Illumina), 2.5 µL of Tn5 Transposase enzyme (Illumina), 16.5 µL of PBS, 0.5 µL of 1% Digitonin (Promega), 0.5 µL of 10% Tween-20 (Sigma-Roche), 5 µL of H_2O). The mixture was then incubated at 37°C for 30 minutes in a heated block shaking at 1000 RPM. This reaction allowed the Tn5 transposase to simultaneously fragment and tag the chromatin with sequencing adapters.

Transposed fragments were purified by using the MinElute Reaction Cleanup Kit (QIAGEN) following manufacturer's instructions and eluted in 21 µL of H_2O. The whole eluted product was amplified for 5 cycles (Table 2.5 and 2.6). Pre-amplified library was stored in ice.

Reagent	Volume (µL)

Primer Ad1 25 µM	2.5
Primer Ad2 25 µM	2.5
NEBNext Master Mix 2x (NEB)	25
Transposed Sample	20

PCR reagents for the pre-amplification of transposed fragments

Temperature (°C)	Duration	Cycles
72	5 min	1
98	30 sec	
98	10 sec	5
63	30 sec	
72	1 min	
4	∞	

PCR conditions for the pre-amplification of transposed fragments

To determine the number of additional cycles needed for an optimal library amplification, 5 µL of pre-amplified library were used to set up a RT-qPCR reaction (Table 2.7 and 2.8). Linear relative fluoresce values were plotted against cycles, and the number of additional cycles needed was defined as the cycle number corresponding to 1/3 of the maximum fluorescence intensity (7).

Reagent	Volume (µL)
Sterile H_2O	3.76
Primer Ad1 25 µM	0.5
Primer Ad2 25 µM	0.5
SYBR Green 25x _(in DMSO)	0.24
NEBNext Master Mix 2x (NEB)	25
Transposed Sample	20

Setup of the RT-qPCR to determine the number of additional cycles of ATAC library amplification

**Temperature (°C)**	**Duration**	**Cycles**
98	30 sec	1
98	10 sec	20
63	30 sec	
72	1 min	
4	∞	

RT-qPCR conditions to determine the number of additional cycles of ATAC library amplification

The remainder of pre-amplified library was amplified for the additional cycles required following the condition in table 2.8. The reaction was purified using the QIAquick PCR Purification Kit (QIAGEN) following manufacturer's instructions. Briefly, the sample was mixed with 5 volumes of Buffer PB and loaded onto a MinElute column (QUIAGEN). The column was centrifuged and washed with 750 µL of Buffer PE. Two sequential centrifugations were performed to remove any residual ethanol. All centrifugation steps were performed at 17900 x g for 1 min. Libraries were eluted in 20 µL of H_2O and, to avoid adapter contamination, further purified by adding 1.2x volumes of AMPure XP beads (Beckman Coulter) following manufacturer's instructions. The libraries were then eluted in 20 µL of H_2O.

Chromatin Immunoprecipitation followed by sequencing (ChIP-seq)

Up to $2x10^7$ cells (depending on the antibody used and the expression level of the TF of interest) were washed 3 times in PBS and resuspended in 10 mL of PBS. To achieve protein-protein cross-linking, cells were incubated with 0.25M Di(N-succinimidyl) glutarate (DSG) (Sigma-Aldrich) for 45 min on rotation at RT. Cross-linked cells were pelleted and washed 3 times with 10 mL of PBS, spinning at 300xg for 5 min at 4°C between washes.

For single crosslinking the pellet was resuspended in 10 mL of PBS. Proteins were fixed to chromatin by adding 16% formaldehyde (Pierce) to a final 1% concentration and incubating on rotation for 10 min at RT. To quench the cross-linking reaction, cells were incubated on ice

for 5 min with 0.4M glycine (Sigma-Aldrich). Cross-linked cells were pelleted and washed twice with 50 mL of ice-cold PBS, spinning at 300xg for 5 min at 4°C between washes.

Cell pellets were resuspended in 10 mL of ice-cold lysis buffer A (10 mM HEPES pH 8.0, 10 mM EDTA pH 8.0, 0.5 mM MEGTA pH 8.0, 0.25% Triton-X 100) with the addition of 1x Protein Inhibitor Cocktail (PIC) (Sigma-Aldrich) and incubated on rotation for 10 min at 4°C. Residual nuclei were pelleted at 500xg for 10 min at 4°C, resuspended in 10 mL of ice-cold Buffer B (10 mM HEPES pH 8.0, 200 mM NaCl, 1 mM EDTA pH 8.0, 0.5 mM MEGTA pH 8.0, 0.01% Triton-X 100) with the addition of 0.1x PIC and incubated on rotation for 10 min at 4°C. Chromatin was pelleted at 500xg for 5 min at 4°C and resuspended to a final concentration of $5x10^6$ cells / 300 µL of ice-cold IP Buffer I (25 mM Tris-HCl, pH 8.0, 150 mM NaCl, 2 mM EDTA, pH 8.0, 1% Triton-X 100, 0.25% SDS) with the addition of 0.1x PIC.
A maximum of 300 µL of chromatin suspension were aliquoted in Bioruptor tubes (Diagenode) and fragmented by using a Bioruptor Pico Sonicator (Diagenode). For each cell type, the number of sonication cycles was optimized to obtain chromatin fragment ranging between 200 and 1000 bp. Each cycle was 30 sec long with a 30 sec break. Sonicated chromatin was spun down at 16000xg for 15 min at 4°C and resuspended in 2 volumes of IP Buffer II (25 mM Tris-HCl, pH 8.0, 150 mM NaCl, 2 mM EDTA, pH 8.0, 1% Triton-X 100, 7.5% Glycerol) with the addition of 1x PIC. Separated reactions were pooled and 10% of the chromatin suspension was stored as input control.

The following IP protocol is optimized for a maximum input of $5x10^6$ cells, and multiple reactions were set up for experiments requiring a greater number of cells. For each IP, 10 µL of Dynabeads Protein G (Thermo Fisher) were washed once with 200 µL of 0.1M citrate phosphate pH 5.0 and twice with 200 µL of 100 mM NaH_2 pH 8.0 phosphate buffer. 4 µg of C/EBPα antibody (sc-1662587 or sc-61, Santa Cruz), RUNX1 antibody (Ab23980) or c-Fos antibody (MA5-15055, Invitrogen) were added to 5 µL of 100 mM NaH_2 pH 8.0 phosphate buffer, 0.5% BSA and incubated with Dynabeads Protein G on rotation for 2 hours at 4°C.

Chromatin was incubated with the antibody-beads mixture for 4 h (C/EBPα and RUNX-1 IPs) or overnight (c-FOS IPs) on rotation at 4°C. Beads were washed once with Wash Buffer I (20 mM Tris-HCl, pH 8.0, 150 mM NaCl, 2mM EDTA pH 8.0, 1% Triton-X 100, 0.1% SDS), twice with

Wash Buffer II (20 mM Tris-HCl, pH 8.0, 500 mM NaCl, 2mM EDTA pH 8.0, 1% Triton-X 100, 0.1% SDS), once with Lithium Chloride (LiCl) Buffer (10 mM Tris-HCl, pH 8.0, 250 mM LiCl, 1mM EDTA pH 8.0, 0.5% NP-40, 0.5% Na-Deoxycholate) and twice with TE/NaCl Buffer (10 mM Tris-HCl, pH 8.0, 50 mM NaCl, 1 mM EDTA pH 8.0). Chromatin was eluted from beads in 100 µL of freshly prepared Elution Buffer (EB) (100 mM $NaHCO_3$, 1% SDS) incubating on a shaker for 30 minutes at RT. Samples, including the input control, were incubated overnight at 65°C in 500 mM NaCl and 500 µg/ml proteinase K (Roche) to reverse crosslinks and digest proteins. After this step, multiple reactions were pooled together.

For quality control, samples were incubated with 1.8x volumes of AMPure XP beads (Beckman Coulter) for 30 minutes at RT. After washing beads twice with 200 µL of ethanol, chromatin was eluted in 50 µL of 0.1x TE pH 8.0.
The quality of the ChIP was assessed by qPCR. The immunoprecipitated DNA and the input control were amplified using primers specific for both putative binding sites of the TF in exam as well as non-bounding sites (Table 2.8) and the enrichment of the target sequences was measured over the input.

**Primer Target**	**Sequence**
Chr18 Forward	ACTCCCCTTTCATGCTTCTG
Chr18 Reverse	AGGTCCCAGGACATATCCATT
IVL Forward	GCCGTGCTTTGGAGTTCTTA
IVL Reverse	CCTCTGCTGCTGCCACTT
PU.1 -14kb Enhancer Forward	AACAGGAAGCGCCCAGTCA
PU.1 -14kb Enhancer Reverse	TGTGCGGTGCCTGTGGTAAT
LAT2 Forward	AAACCCAGAACAACCCAGGC
LAT2 Reverse	ATGAGGAAGGATGTGTGTGCGG
IGFBP7 Forward	GTCAAGCACTAAAAGGACAAACCG
IGFBP7 Reverse	TGAATGCCACTGGGAG

List of primers used to assess ChIP quality

ChIP-seq library preparation

ChIP library preparation was performed by using the KAPA Hyper Prep Kit (Roche) as described for DNasI-Seq, except for a 200-450 bp library size selection.

DNase I-seq and ATAC-seq data analysis

Sequencing adapters and low-quality reads and were removed using Trimmomatic (8). Reads were then mapped to the human genome version hg38 using Bowtie v2.2.3 (9), and PCR duplicated reads were removed using Picard (http://broadinstitute.github.io/picard). Only reads aligned to unique genomic positions were retained using Samtools. The bamCoverage function from BEDTools (10) was used to generate read density profiles that were then visualized using the UCSC Genome Browser (11). DHSs, correspond to regions of open chromatin (peaks) were identified using MACS2 (12). Peaks included in the ENCODE hg38 blacklist (13) were removed. Peaks were annotated to their associated gene using Promoter Capture Hi-C. Where a peak did not occur in a restriction fragment and therefore could not be mapped to a gene promoter by Hi-C, the peak was annotated to the closest gene using the annotatePeaks.pl function in Homer (14). Peaks were further annotated as promoter proximal if within -/+2 kb of a gene transcription start site (TSS) and were classified as distal otherwise. If two peaks had summits within 200 bp of each other, these peaks were combined into a single peak with a new summit position defined as the mid-point between the summits of the original peaks.

To maximise the precision and accuracy of peak detection, a reference peak set was created using a merged dataset of all sequencing experiments from all primary samples. To do this, the aligned reads (bam files) of all the samples were merged into a single alignment using the merge function in samtools. Peaks were then identified using this merged alignment using MACS2 as described in section 2.2.31, and these peak positions were used as the reference peak position in all further downstream analysis. Tag counts were retrieved from these peaks using featureCounts (15). Counts were then normalized as count per million (CPM) using the DEseq2 (16) package in R and then further quantile normalized using quantile normalization.

AML subtype-specific hypersensitive sites analysis

To identify genomic regions of differential chromatin accessibility specifically enriched in *CEBPA*$^{N/C}$, t(8;21) or PBSC blasts, the average CPM values for each hypersensitive site within

each group were calculated and further log2-transformed as log2(CPM +1). A genomic site was considered to be differentially accessible if the fold-difference of the CPM between two subtype was greater than

Clustering of chromatin accessibility data from primary samples

To generate the heatmap in Fig. 1C, the Pearson correlation coefficients for each pair of samples were calculated using the log2 transformed CPM values. The resulting correlation matrix was then hierarchically clustered using Euclidean distance with complete linkage clustering in R

Low-quality reads and sequencing adapters were removed using Trimmomatic (8) and aligned to the human genome version hg38 using HISAT2 (17). StringTie (18) was used to assemble reads into transcripts and calculate gene expression values as fragment per kilobase of transcript per million fragments (FPKM). Gene models from the Ensembl database were used as reference transcriptome (19). Only genes that were considered expressed with a FPKM > 1 in at least one sample were retained for further analysis. Values were log2-transformed as log2(FPKM +1

Clustering of RNA-seq data from primary samples

To generate the heatmap in Fig. 1B, the Pearson correlation coefficients for each pair of samples were calculated using the log2 transformed FPKM values and hierarchically clustered using Euclidean distance with complete linkage clustering in R

Differential gene expression analyses

Gene expression was initially measured as number of reads mapped to a transcript with featureCounts (15) using the hg38 assembly from the Ensembl database as reference transcriptome (19) (-p -s 2 parameters). Counts were filtered and normalized to Counts Per Million (CPM) using EdgeR (20). Differential gene expression analysis were carried out using the Limma package (21) in R. A gene was considered as differentially expressed between two conditions if it had a fold-change greater than 2 and a Benjamini-Hochberg adjusted p-value < 0.05. For primary samples analyses, samples within the PBSC, $\textit{CEBPA}^{N/C}$ and t(8;21) blast groups were treated as biological replicates

KEGG pathway analysis

Kyoto Encyclopaedia for Genes and Genomes (KEGG) pathway enrichment analysis were performed using the ClueGo v3.8.2 plugin (22) for Cytoscape v2.5.7 (23) using a right-sided hypergeometric test with Benjamini-Hochberg adjusted p-value for multiple testing. A pathway was considered significantly enriched if the adjusted p-value was < 0.05.

Promoter Capture Hi-C data analysis

Promoter Capture Hi-C data were processed with the HiCUP pipeline (24). The paired-end sequencing reads, called di-tag, were separately mapped to the hg38 genome, repaired and filtered for experimental artifact (i.e., self-ligation and re-ligation products) and PCR duplicates. Mapped di-tags were then analysed with GOTHiC (25). This package uses a cumulative binomial test to assign a p-value to each interaction and detects di-tags significantly enriched compared to a background model of random interactions. The HOMER software package was used to determine statistically significant interactions, taking into account the p-value and false discovery rate (FDR) relative to a background model.

***CEBPA*$^{N/C}$-specific gene regulatory network construction**

Motif search within hypersensitive sites specific for *CEBPA*$^{N/C}$ and PBSCs was performed using the findMotifGenome.pl function in HOMER (14). Sites were linked to their associated gene promoters using Chi-C data where possible, or otherwise to the nearest expressed gene. The number of motifs was counted for each hypothetical target gene, and the significance of motif enrichment was determined by bootstrapping analyses. To do this, the number of transcription factor motifs in the peak set being examined were counted using the annotatePeaks.pl function in Homer. A random set of peaks equal in size to the peak set being examined was then randomly sampled from the complete set of peaks across all patients. This procedure was repeated 1000 times and produced a distribution of expected motif counts. The mean (μ) and standard deviation (σ) of this distribution was then calculated, and were then used to calculate a Z-score as

$$z = \frac{x - \mu}{\sigma}$$

where x is the number of motifs in the actual peak set. Here, a positive Z-score suggests that the number of motifs in the peak set is greater than what could be expected by chance, representing an enrichment of that motif. Transcription factor family-specific motifs with positive Z-score values selected for further analysis and were then linked to their putative target genes using either CHi-C or closest gene as described above in section 2.2.35.

ChIP-Seq data analysis

Raw sequencing reads were trimmed to remove low-quality sequences and adaptors using Trimmomatic. Processed reads were then aligned to the human genome version hg38 using Bowtie2 v2.2.3, with only uniquely aligned reads retained for further analysis. PCR duplicated reads were identified and removed from the alignments using the MarkDuplicates function in Picards tools. Peaks were called using MACS2 with default settings. The resulting peaks were then filtered using the hg38 Blacklist from ENCODE to remove potential artefacts from the data. Only peaks that were found within open chromatin regions as defined by ATAC-Seq data were retained. Peaks were then annotated to their target genes based on the Chi-C data were available or by closest gene using the annotatePeaks.pl function in Homer

Motif co-localization analysis

The genomic co-ordinates for each transcription factor binding motifs were retrieved from within the KO52 ATAC-Seq peaks that were found to be bound by C/EBPα, FOS and RUNX1 using the annotatePeaks.pl function in Homer. Motif co-occurrence was then measured by counting the number of times a pair of motifs were found within 50bp of each other in this peak set. To measure the significance of this co-occurrence, we next applied a re-sampling analysis whereby we sampled a random set of ATAC peaks from the set of all ATAC peaks found in KO52 cells. The number of motif pairs in this random set was then counted. This procedure was repeated 1000 times and produced a distribution of co-occurrence counts for each pair of motifs. A Z-score could then be computed for each pair as

$$z = \frac{x - \mu}{\sigma}$$

where x is the number of motif pairs in the C/EBPα, FOS and RUNX1 bound sites, μ is the average number of motif pairs in 1000 random peak sets and σ is the standard deviation. The

resulting z-score matrix was then hierarchically clustered using complete linkage clustering of the Euclidean distance in R and displayed as a heatmap.

Single Cell RNA-Seq data analysis

Reads from single-cell RNA-Seq experiments were aligned to the human genome (version hg38) and quantified using the count function in CellRanger v3.1.0 from 10x Genomics and using gene models from Ensembl as the reference transcriptome. Unique Molecular Identifier (UMI) count data was filtered for low quality cells by removing cells with less than 500 and more than 5000 detectable genes. Cells that had more than 15% of UMIs aligned to mitochondrial transcripts were also excluded from further analysis. UMI counts were normalized using the log-normalize method in the Seurat package v3.2.2 (26) in R v3.6.1. The cell cycle stage was then estimated for each cell using the CellCycleScoring function in Seurat and using the in-built lists of cell cycle stage specific genes. To account for the possible effect of cell cycle stage on downstream clustering analysis, S-phase and G2M-phase scores were included as variables in a linear regression model using the ScaleData function in Seurat. Principal Components Analysis (PCA) was then performed on the normalized and scaled data, with the first 25 principal components selected for further analysis. Cells were then clustered using the FindClusters function in Seurat and visualized using Uniform Manifold Approximation and Projection (UMAP). Cluster marker genes, corresponding to genes that are significantly higher expressed in a cluster compared to all other cells outside of that cluster, were identified using the FindAllMarkers function. Genes that had an average log2-fold change of at least 0.25 with an adjusted p-value less than 0.05 were selected as marker genes.

In order to classify a single-cell cluster as either blast or LSC, we first identified blast and LSC specific gene expression signatures using bulk RNA-Seq from sorted cell populations. To do this, raw paired-end reads from bulk RNA-Seq experiments were trimmed to remove low-quality reads and sequencing adaptors using Trimmomatic v0.39 (8). Processed reads were then aligned to the human genome (version hg38) using Hisat2 v2.1.0 (17). Gene expression was then measured as Fragments Per Kilobase of transcript per Million mapped reads (FPKM) using Stringtie v2.1.1 (18). Only genes that were considered expressed with an FPKM greater than or equal to 1 in either LSCs or blasts were retained for further analysis. Gene expression

values were normalized using upper-quartile normalization and further log2-transformed as log2(FPKM + 1). Genes were then ranked according to the log2-fold difference between blasts and LSCs. This ranked list could then be used as a reference gene expression signature for Gene Set Enrichment Analysis (GSEA).

GSEA was carried out using the fgsea package v1.10.1 (27) in R. To do this, cluster marker genes from single-cell clusters were used as pathways and compared to the gene expression signatures derived from the bulk data. This analysis produced a Normalized Enrichment Score (NES) for each cluster, with a positive NES suggesting that a cluster has a more blast-like gene expression signature and a negative NES suggesting a more LSC-like signature. Only clusters with a Benjamini-Hochberg adjusted p-value < 0.1 were considered to be positively classified as either LSC or blast.

Single-cell trajectory analysis was carried out using Monocle3 v0.2.3 (28). Processed data from Seurat was imported to Monocle and trajectories were inferred using the learn_graph command. Pseudotime was then calculated using the order_cells command, using cells from the earliest inferred LSC population (LSC-1). Trajectories were then plotted on the UMAP calculated by Seurat.

Supplementary references:

1. Ng SW, Mitchell A, Kennedy JA, Chen WC, McLeod J, Ibrahimova N, et al. A 17-gene stemness score for rapid determination of risk in acute leukaemia. Nature. 2016;540(7633):433-7.
2. Olive M, Williams SC, Dezan C, Johnson PF, Vinson C. Design of a C/EBP-specific, dominant-negative bZIP protein with both inhibitory and gain-of-function properties. J Biol Chem. 1996;271(4):2040-7.
3. Martinez-Soria N, McKenzie L, Draper J, Ptasinska A, Issa H, Potluri S, et al. The Oncogenic Transcription Factor RUNX1/ETO Corrupts Cell Cycle Regulation to Drive Leukemic Transformation. Cancer Cell. 2018;34(4):626-42 e8.
4. Adams FF, Heckl D, Hoffmann T, Talbot SR, Kloos A, Thol F, et al. An optimized lentiviral vector system for conditional RNAi and efficient cloning of microRNA embedded short hairpin RNA libraries. Biomaterials. 2017;139:102-15.
5. Illendula A, Gilmour J, Grembecka J, Tirumala VSS, Boulton A, Kuntimaddi A, et al. Small Molecule Inhibitor of CBFbeta-RUNX Binding for RUNX Transcription Factor Driven Cancers. EBioMedicine. 2016;8:117-31.

6. Corces MR, Trevino AE, Hamilton EG, Greenside PG, Sinnott-Armstrong NA, Vesuna S, et al. An improved ATAC-seq protocol reduces background and enables interrogation of frozen tissues. Nat Methods. 2017;14(10):959-62.
7. Buenrostro JD, Wu B, Chang HY, Greenleaf WJ. ATAC-seq: A Method for Assaying Chromatin Accessibility Genome-Wide. Curr Protoc Mol Biol. 2015;109:21 9 1- 9 9.
8. Bolger AM, Lohse M, Usadel B. Trimmomatic: a flexible trimmer for Illumina sequence data. Bioinformatics. 2014;30(15):2114-20.
9. Langmead B, Salzberg SL. Fast gapped-read alignment with Bowtie 2. Nat Methods. 2012;9(4):357-9.
10. Quinlan AR, Hall IM. BEDTools: a flexible suite of utilities for comparing genomic features. Bioinformatics. 2010;26(6):841-2.
11. Kent WJ, Sugnet CW, Furey TS, Roskin KM, Pringle TH, Zahler AM, et al. The human genome browser at UCSC. Genome Res. 2002;12(6):996-1006.
12. Zhang Y, Liu T, Meyer CA, Eeckhoute J, Johnson DS, Bernstein BE, et al. Model-based analysis of ChIP-Seq (MACS). Genome Biol. 2008;9(9):R137.
13. Amemiya HM, Kundaje A, Boyle AP. The ENCODE Blacklist: Identification of Problematic Regions of the Genome. Sci Rep. 2019;9(1):9354.
14. Ziemann M, Kaspi A, Lazarus R, El-Osta A. Motif analysis in DNAse hypersensitivity regions uncovers distal cis elements associated with gene expression. Bioinformation. 2013;9(4):212-5.
15. Liao Y, Smyth GK, Shi W. featureCounts: an efficient general purpose program for assigning sequence reads to genomic features. Bioinformatics. 2014;30(7):923-30.
16. Love MI, Huber W, Anders S. Moderated estimation of fold change and dispersion for RNA-seq data with DESeq2. Genome Biol. 2014;15(12):550.
17. Kim D, Langmead B, Salzberg SL. HISAT: a fast spliced aligner with low memory requirements. Nat Methods. 2015;12(4):357-60.
18. Pertea M, Pertea GM, Antonescu CM, Chang TC, Mendell JT, Salzberg SL. StringTie enables improved reconstruction of a transcriptome from RNA-seq reads. Nat Biotechnol. 2015;33(3):290-5.
19. Howe KL, Achuthan P, Allen J, Allen J, Alvarez-Jarreta J, Amode MR, et al. Ensembl 2021. Nucleic Acids Res. 2021;49(D1):D884-D91.
20. Robinson MD, McCarthy DJ, Smyth GK. edgeR: a Bioconductor package for differential expression analysis of digital gene expression data. Bioinformatics. 2010;26(1):139-40.
21. Law CW, Alhamdoosh M, Su S, Dong X, Tian L, Smyth GK, et al. RNA-seq analysis is easy as 1-2-3 with limma, Glimma and edgeR. F1000Res. 2016;5.
22. Bindea G, Mlecnik B, Hackl H, Charoentong P, Tosolini M, Kirilovsky A, et al. ClueGO: a Cytoscape plug-in to decipher functionally grouped gene ontology and pathway annotation networks. Bioinformatics. 2009;25(8):1091-3.
23. Shannon P, Markiel A, Ozier O, Baliga NS, Wang JT, Ramage D, et al. Cytoscape: a software environment for integrated models of biomolecular interaction networks. Genome Res. 2003;13(11):2498-504.
24. Wingett S, Ewels P, Furlan-Magaril M, Nagano T, Schoenfelder S, Fraser P, et al. HiCUP: pipeline for mapping and processing Hi-C data. F1000Res. 2015;4:1310.
25. Mifsud B, Martincorena I, Darbo E, Sugar R, Schoenfelder S, Fraser P, et al. GOTHiC, a probabilistic model to resolve complex biases and to identify real interactions in Hi-C data. PLoS One. 2017;12(4):e0174744.

26. Stuart T, Butler A, Hoffman P, Hafemeister C, Papalexi E, Mauck WM, 3rd, et al. Comprehensive Integration of Single-Cell Data. Cell. 2019;177(7):1888-902 e21.
27. Korotkevich G, Sukhov V, Budin N, Shpak B, Artyomov MN, Sergushichev A. Fast gene set enrichment analysis. bioRxiv. 2021:060012.
28. Trapnell C, Cacchiarelli D, Grimsby J, Pokharel P, Li S, Morse M, et al. The dynamics and regulators of cell fate decisions are revealed by pseudotemporal ordering of single cells. Nat Biotechnol. 2014;32(4):381-6.
